# Observation of chiral emission enabled by collective guided resonances

**DOI:** 10.1038/s41565-025-01964-7

**Published:** 2025-07-01

**Authors:** Ye Chen, Mingjin Wang, Jiahao Si, Zixuan Zhang, Xuefan Yin, Jingxuan Chen, Nianyuan Lv, Chenyan Tang, Wanhua Zheng, Yuri Kivshar, Chao Peng

**Affiliations:** 1 State Key Laboratory of Photonics and Communications, School of Electronics & Frontiers Science Center for Nano-optoelectronics, Peking University, Beijing, China; 2 Laboratory of Solid State Optoelectronics Information Technology, Institute of Semiconductors, CAS, Beijing, China; 3 Nonlinear Physics Centre, Research School of Physics, Australian National University, Canberra, Australian Capital Territory Australia; 4 Center of Materials Science and Optoelectronics Engineering, University of Chinese Academy of Sciences, Beijing, China; 5 Hangzhou Institute for Advanced Study, University of Chinese Academy of Sciences, Hangzhou, China; 6 College of Future Technology, University of Chinese Academy of Sciences, Beijing, China; 7 Peng Cheng Laboratory, Shenzhen, China

**Keywords:** Photonic crystals, Semiconductor lasers

## Abstract

A simple yet insightful question is whether it is possible to arrange optical resonances in such a way that their collective response differs from that of the individual constituents. Here, inspired by the collective oscillation of spatially localized modes and Fourier duality between real and momentum spaces, we demonstrate a chiral emission of collective guided modes by leveraging the omnidirectional hybridization of individual guided resonances within a photonic crystal slab. Specifically, we encircle a uniform photonic crystal with isotropic boundaries and hybridize discrete bulk guided resonances into a series of collective modes owing to the scatterings of the boundaries. This results in a chiral spiral vortex emission in real space. By using asymmetric pumping to lift the chiral symmetry, we then achieve stable single-mode lasing oscillation of the spiral collective mode and confirm the nature of vortex emission through polarization-resolved imaging and self-interference patterns, thus demonstrating a vivid example of collective oscillations in the momentum space.

## Abstract (web summary)

Chiral emission from collective guided modes is demonstrated within a photonic crystal slab, showing an example of collective oscillations in the momentum space.

## Main

Collective oscillation refers to a cluster of individual resonators collectively oscillating as a whole but exhibiting characteristics distinct from those of the individual resonators. This phenomenon has been observed in photonic^1–3^, plasmonic^4–6^ and quantum^7,8^ wave systems, and was captured by the famous remark, ‘more is different’, by P. W. Anderson in many-body wave systems^9^. An example of collective oscillation is the Dicke state, a coherent collection of atoms giving rise to the phenomenon of superradiance^10,11^. In plasmonics, nanoparticles arranged in a regular lattice (chains^1,4–6,12,13^, gratings^14^, arrays of nanoholes^15^ and periodic assemblies^16^) can exhibit collective lattice resonances leading to higher-quality factors (*Q*s) than their constituents. These collective behaviours originate from the interaction between one spatially localized resonance and its two or more nearby neighbours in real space. However, considering that the real and momentum spaces are closely related by Fourier duality, we anticipate that the interactions between discrete bulk resonances may lead to a distinct class of collective oscillations in momentum space. Specifically, it has been recognized that guided resonances (GRs) in photonic crystal (PhC) slabs possess discrete wavevectors owing to the translational invariance of periodic structures, exhibiting a wave nature that extends in real space^17^. By effectively coupling them, the GRs can oscillate in unison, creating collective guided resonances (CGRs) with more complex spatial characteristics and radiation patterns than those of basic plane waves. We expect that these CGRs will uncover rich and exotic phenomena, which, however, remain to be explored.

Our goal is to construct a vortex beam in real space with chiral emission as an example of nontrivial CGRs. Optical vortices carry a spiral phase wavefront with quantized orbital angular momentum^18^. Owing to the unique ability to spatially differentiate photons, optical vortices find applications in sensing^19^, micromanipulation^20,21^ and optical communication^22–25^. Vortex emission has been observed in momentum space by utilizing polarization vortex carried by bound states in the continuum (BICs)^26–28^ or using photonic disclination to modify the polarization orientations of GRs^29^, both of which contribute azimuthal gradual Pancharatnam–Berry phases. However, to generate optical vortices in real space, planar geometries that either bend light paths or create spiral phase fronts appear essential. These demonstrations align with the examples of angular-grating incorporated microrings^30–34^ and vertical-cavity surface-emitting lasers with a spiral phase plate^35–37^. Although the GRs are extending waves without explicitly defined light paths, their collective oscillation may exhibit chiral emission. Recall the simple yet nontrivial fact that in a Fabry–Pérot cavity, the counter-propagating plane waves are coupled by the reflective mirrors to create a ‘collective’ mode. Similarly, we propose that arranging geometric boundaries of GRs can introduce couplings between them, thereby leading to the chiral CGR that we aim to achieve.

In this work, we demonstrate chiral lasing emission by utilizing the collective oscillation of GRs in PhC slabs, in which optical modes circulate in real space and radiate as spiral phase fronts. Specifically, we design a square-lattice PhC encircled by a circular lateral boundary on an active InGaAsP membrane. Owing to the circular boundary’s scattering, the GRs that originally propagated along specific Bloch wavevectors omnidirectionally hybridize into twofold degenerate collective modes with opposite chirality. We then apply the quasi-BICs conditions to enhance the *Q*s and use the technique of asymmetric pumping to break chiral symmetry. As a result, we experimentally achieve stable single-mode lasing oscillation of the collective mode, which exhibits chiral emission in real space, as verified through polarization-resolved imaging and self-interference patterns.

## Principle and design

**Fig. 1:**
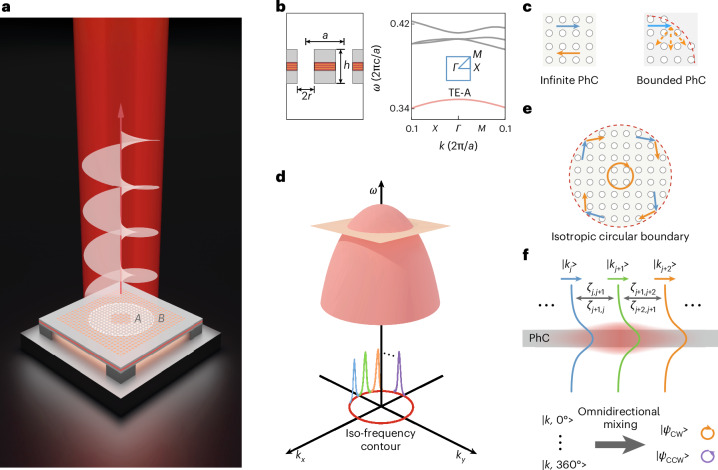
Principle of collective modes. **a**, Schematic of collective lasing under optical pumping. The central PhC region A is surrounded by a heterogeneous PhC region B with a circular boundary. **b**, The PhC geometry is defined by lattice constant *a*, air-hole radius *r* and slab thickness *h* (left panel), which gives rise to a band structure accordingly (right panel), where the TE-A band of region A near the 2nd-*Γ* point is embedded in the band gap of region B. **c**, The principle of boundary scattering. The Bloch waves propagate along straight directions in an infinite periodic PhC (left panel), while the boundary scatterings give additional momenta Δ*k* to alter their directions (right panel). **d**, A 3D visualization of the TE-A band’s dispersion, showing an isotropic iso-frequency contour at the Brillouin zone centre to support the collective modes. **e**, The scattering process in real space upon the isotropic circular boundary. **f**, The omnidirectional mixing of Bloch waves in momentum space results in twofold degenerate modes $\left\vert {\psi }_{\mathrm{CW;CCW}}\right\rangle$. Source data

We schematically present our design in Fig. 1a, in which square-lattice air holes are patterned on a suspended InGaAsP membrane. Six layers of multiple quantum wells are embedded in the membrane centre as optical gain materials at the C-band of the telecom wavelength. We use the photonic band gap to localize the light in the transverse direction^38^. That is, the central region (region A with radius *r*_A_) is surrounded by a heterogeneous PhC (region B with radius *r*_B_) to form a circular boundary of the photonic band gap for lateral confinement. The details of the epitaxial wafer and cavity are presented in Supplementary Section 1. Then, the scatterings at the circular boundary would drive the bulk GRs that are aligned with the iso-frequency contour in momentum space to collectively oscillate. As a result, we found a set of collective modes that counter-intuitively circulate in real space, enabling a spiral phase wavefront upon radiation in the out-of-plane direction.

To elaborate on the principle of collective oscillation, we first present the detailed PhC geometry and band diagram (Fig. 1b), focusing on the transverse electric band A (TE-A) of region A near the second-order *Γ* point. It should be noted that, in infinite periodic PhCs, two bulk GRs $\left\vert k\right\rangle$ and $\left\vert {k}^{{\prime} }\right\rangle$ with Bloch wavevectors $k\ne {k}^{{\prime} }$ are mutually orthogonal. Consequently, they propagate independently in straight paths along specified directions owing to the PhC’s translational symmetry (left panel, Fig. 1c), preventing them from altering propagation directions to create a well-defined closed loop in real space. However, a nonuniform PhC boundary can generate additional momenta $\Delta k=k-{k}^{{\prime} }$ (refs. ^39,40^), leading to the coupling between $\left\vert k\right\rangle$ and $\left\vert {k}^{{\prime} }\right\rangle$ (right panel, Fig. 1c). Essentially, the modes confined by the circular boundary can be viewed as a collective linear combination of bulk GRs owing to the boundary’s scattering (we denote them as CGRs), allowing for well-defined energy flows in the real space to generate unique phase fronts in the radiation (Fig. 1e).

Specifically, we present a three-dimensional (3D) visualization of TE-A band’s dispersion in Fig. 1d, revealing a quadratic curvature near the *Γ* point. To ensure the temporal stability of collective oscillation, the GRs involved in the linear combination must possess identical frequencies, namely they should align with the iso-frequency contour of the band. As depicted in Fig. 1d (red circle), this contour is nearly circular with a radius of $\Delta {k}_{r}={\mu }_{m}^{l}/R$, where ${\mu }_{m}^{l}$ denotes the *l*th zero of the *m*th Bessel function ${J}_{m}(\;{\mu }_{m}^{l})=0$ and *R* denotes the cavity’s geometrical radius. When *R* is sufficiently large, the real-space boundary tends towards roundness regardless of the PhC discreteness. At the same time, the momentum space iso-contour shrinks, resulting in nearly isotropic dispersion. For our design featuring *R* = 10*a*, we observe that both the boundary and dispersion remain almost invariant under the rotations of any angle. Consequently, the bulk GRs (coloured curves) ranging from $\left\vert k,{0}^{\circ }\right\rangle$ to $\left\vert k,36{0}^{\circ }\right\rangle$ on the iso-contour would propagate and scatter omnidirectionally, as illustrated in Fig. 1e for real space and Fig. 1f for momentum space.

We further elaborate on the momentum scattering process (Fig. 1f), where a bulk GR, $\left\vert {k}_{j}\right\rangle$, characterized by given in-plane wavevector with a defined vertical profile, couples with its neighbouring GRs ($\cdots \,\left\vert {k}_{j-2}\right\rangle ,\left\vert {k}_{j-1}\right\rangle ,\left\vert {k}_{j+1}\right\rangle ,\left\vert {k}_{j+2}\right\rangle \,\cdots \,$) along the iso-contour governed by coupling strengths *ζ*_*ij*_. The underlying physics of this system is analogous to that of an array of nanoparticles, where spatially localized resonances interact with neighbouring particles and create non-local collective oscillations in real space with compact wave packets in momentum space. According to Fourier duality, real and momentum spaces are linked as a Fourier pair. Consequently, the couplings between discrete GRs $\left\vert {k}_{j}\right\rangle$ in momentum space should also hybridize them into CGRs $\left\vert \psi \right\rangle ={\sum }_{j}{a}_{j}\left\vert {k}_{j}\right\rangle$ with unique patterns in real space. In our design, these CGRs should preserve continuous rotational symmetry similar to that of a microring, resulting in degenerate paired modes that rotate clockwise (CW) and counterclockwise (CCW) in real space without physical light paths. Moreover, a spiral phase front is imparted to the emitted radiation through the PhC’s diffraction as a vortex beam. More discussions are presented in Supplementary Section 2.

**Fig. 2:**
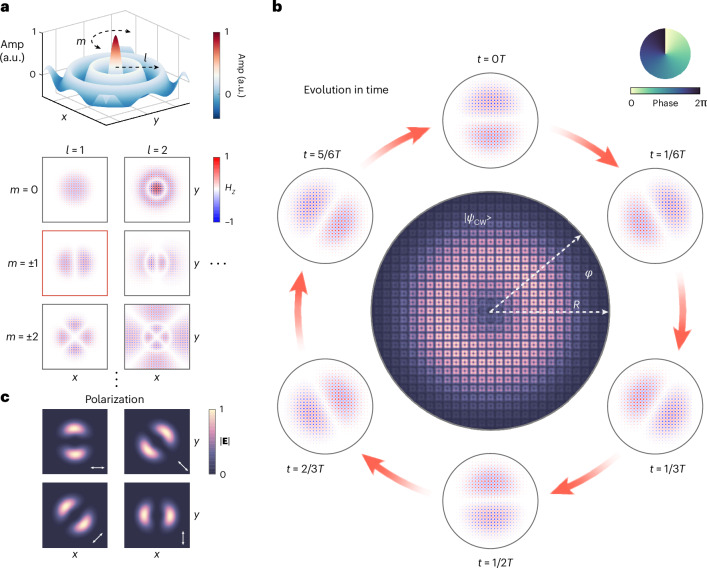
Spiral phase in real space. **a**, The amplitudes (Amp) of collective modes in our circular PhC cavity are Bessel functions *J*_*m*_(*r*) with quantum numbers *m* and *l* in the azimuth and radial directions, respectively (upper panel), and their *H*_*z*_ field distributions are plotted (lower panel). **b**, As the candidate for lasing oscillation, the field strength (∣**E**∣) of collective mode (1, 1) is illustrated as a donut pattern. Moreover, the snapshots of magnetic fields *H*_*z*_ at a sequence of time intervals in one oscillation cycle (0, *T*) show the temporal rotating of the mode that generates a spiral phase from 0 to 2π. **c**, The polarization-solved distribution of collective mode (1, 1) exhibits two lobes with equal density intensities as a distinct feature of the vortex beam.

The CGRs are identified through numerical simulation (COMSOL Multiphysics; Fig. 2a), consistent with analytical solutions derived from polar coupled wave theory^41,42^ (Supplementary Section 3). Their slow-varying envelopes are in the form of Bessel functions *J*_*m*_(*r*), denoted by quantum numbers *m* and *l* in the azimuth and radial directions, respectively. Notably, for *m* ≠ 0, each quantum number (*m*, *l*) corresponds to a pair of twofold degenerate modes that rotate in either CW or CCW directions. For further analysis, we focus on the CW-rotating mode of (1, 1), which we chose as the lasing candidate. To visualize the rotational motion in real space, we compute the snapshot magnetic field *H*_*z*_ at fixed time intervals within a single oscillation cycle, ranging from *t* = 0 to *t* = *T* (Fig. 2b). This rotation generates a spiral phase spanning from 0 to 2π in out-of-plane radiation, akin to the motion of a propeller driving water. Furthermore, the electrical field strength (∣**E**∣) is plotted in the centre of Fig. 2b, showing a donut pattern in real space, as a consequence of temporal averaging the energy flow. When a linear polarizer is placed before observing the donut beam, two lobes with equal density intensities appear along the normal direction of the polarizer’s major axis, providing a distinct feature of its vortex nature (Fig. 2c). To make the chosen CGR more favourable for lasing, we use off-*Γ* BICs to suppress radiation loss^38,43^. More details are provided in Supplementary Section 4.

**Fig. 3:**
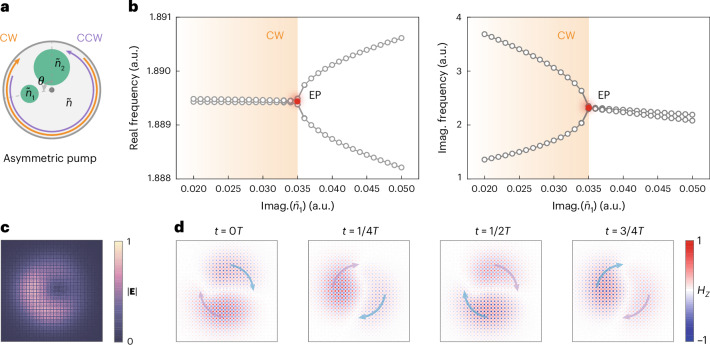
Chiral symmetry breaking. **a**, A simple model of asymmetric pumping for lifting the degeneracy of CW and CCW modes. Two circular pump beams are applied with a relative angle *θ*, while keeping the same distance to the centre of the cavity fixed as 2.5 μm and the radii fixed as 1 μm and 2 μm, respectively. $\tilde{n}$ and ${\tilde{n}}_{1,2}$ are the complex indices of the PhC cavity area, which are unpumped and pumped by two beams, respectively. **b**, The evolution of complex eigenfrequencies in parameter space, showing the emergence of an EP. The parameters are set as $\tilde{n}=3.25-0.01i$, ${\tilde{n}}_{2}=3.25+0.01i$, $Re({\tilde{n}}_{1})=3.25$ and *θ* = 100.15°; the EP is found at $Im({\tilde{n}}_{1})=0.035$, at which the two eigenstates collapse to single CW chirality. The chirality degrades when the parameters deviate from the EP in the region where the real eigenfrequencies are degenerate (orange-shaded), in which the difference in the imaginary parts offers mode selection for single-mode lasing. Imag.(*ñ*_1_) represents the imaginary part of *ñ*_1_. **c**, The electrical field strength ∣**E**∣ of the eigenstate at the EP, exhibiting a donut shape in real space. **d**, The evolution of snapshot magnetic field *H*_*z*_ in a time interval of 1/4*T*, clearly showing the rotation motion along CW direction. All results are calculated from numerical simulations (COMSOL Multiphysics). Source data

Next, we explore how to achieve single-mode lasing by breaking chiral symmetry. For a perfect design with continuous rotational symmetry on the boundary and dispersion, the CW and CCW modes $\left\vert {\psi }_{\mathrm{CW}}\right\rangle$ and $\left\vert {\psi }_{\mathrm{CCW}}\right\rangle$ are twofold degenerate, possessing identical complex eigenfrequencies *Ω*_0_ described by ${{\bf{H}}}_{{\bf{0}}}\left\vert {\psi }_{\mathrm{CW};\mathrm{CCW}}\right\rangle ={\Omega }_{0}\left\vert {\psi }_{\mathrm{CW};\mathrm{CCW}}\right\rangle$, where **H**_**0**_ denotes the system’s Hamiltonian. However, in realistic samples, fabrication imperfections inevitably cause losses and pumping alignment introduces asymmetric gain, thus showing non-Hermitian coupling effects depicted by a perturbed Hamiltonian Δ**H** (Fig. 3a) with off-diagonal terms *κ* and ηκ^*^ to lift the chiral degeneracy of $\left\vert {\psi }_{\mathrm{CW;CCW}}\right\rangle$^44–48^ to create perturbed modes $\left\vert {\psi }_{\mathrm{CW};\,\mathrm{CCW}}^{{\prime} }\right\rangle$.

We introduce a simplified model to demonstrate such chiral symmetry breaking, as the cavity (complex index $\tilde{n}$) is pumped by two circular beams with different positions and sizes to represent asymmetric pumping (Fig. 3a). ${\tilde{n}}_{1,2}$ denotes the indices of pumped area and *θ* denotes the two beams’ relative angle. According to the theory, non-Hermitian coupling would create two complex Riemann sheets in parameter space connected by an exceptional point (EP), at which the two eigenstates collapse to a single chirality. This phenomenon has been confirmed by simulations (COMSOL Multiphysics; Fig. 3b), in which we fix $\tilde{n}=3.25-0.01i$ and ${\tilde{n}}_{2}=3.25+0.01i$ and scan the imaginary part of ${\tilde{n}}_{1}$ from 0.02*i* to 0.05*i*. The EP was found at ${\tilde{n}}_{1}=3.25+0.035i,\theta =100.1{5}^{\circ }$, at which the eigenstate’s electrical field strength exhibits a donut pattern in real space (Fig. 3c). By changing the eigenstate’s phases, a CW rotation motion is identified from the snapshot magnetic fields (Fig. 3d) consistent with Fig. 2b. In theory, the purest chirality appears right at the EP while it gradually degrades when departing it. In particular, in the shaded region in Fig. 3b, where the real eigenfrequencies remain almost degenerate, non-Hermitian couplings create a difference in *Q*s, driving one of $\left\vert {\psi }_{\mathrm{CW};\,\mathrm{CCW}}^{{\prime} }\right\rangle$ prevailing over mode competition for single-mode lasing. Similar phenomena have been reported in microring lasers^30,49^. More discussions are presented in Supplementary Sections 5 and 6.

## Sample fabrication and experimental set-up

**Fig. 4:**
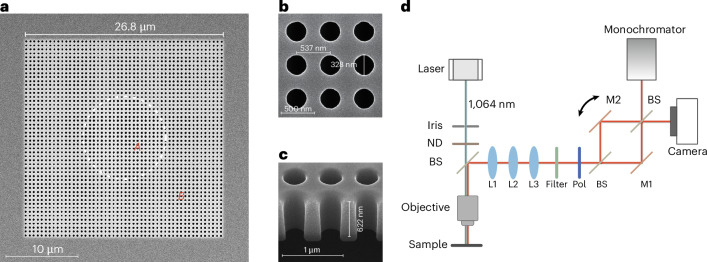
Fabricated sample and measurement set-up. **a**, The scanning electron microscope images of the fabricated sample show the hetero-structures PhC consisting of region A and region B from a top view. **b**, A detailed top view. **c**, A detailed side view of the undercut structure. A focused ion beam is used to cleave the PhC. **d**, Schematic of the experimental set-up. Iris, iris diaphragm; ND, absorptive neutral density; BS, beam splitter; L, lens; Pol, linear polarizer; M, gold-coated mirror.

To verify our principle and design, we fabricate samples from an InGaAsP multiple-quantum-well wafer on an InP substrate. The PhCs are exposed by electron beam lithography, followed by plasma dry etching. We subsequently remove the sacrificial layer by using hydrochloric acid to restore vertical mirror symmetry required by the BICs. The sample is observed by using the scanning electron microscope. The top view (Fig. 4a) shows a total footprint of 26.8 × 26.8 μm^2^, in which regions A and B are separated by the circular dashed line. We further cleave the PhC by focused ion beam, to observe the detailed top and side views (Fig. 4b,c). The structural parameters are characterized as the periodicity *a* = 537 nm, diameter *r*_A_ = 164 nm and height *h* = 622 nm, agreeing well with our design (see Methods and Supplementary Sections 1 and 4 for details).

The measurement set-up is shown in Fig. 4d. The sample is optically pumped at room temperature by using a 1,064 nm pulsed laser with a repetition rate of 10 kHz and a duration of 2 ns. The pump beam is first tailored to be transversely asymmetric by an iris diaphragm, to enable the asymmetric pumping condition, and further attenuated by an absorptive filter with an attenuation ratio of −24 dB to avoid sample damage. The average pump power is measured by a power meter before the filter to minimize power fluctuation and noise at low pump powers.

The sample lases and generates a vertical-emitting vortex beam when sufficient pump energy is focused on its surface by an objective lens (X50). The emitting and reflected beams are collected by the same objective lens and then pass through lens L1 to form real-space imaging, which is further enlarged 6 times by a 4*f* system (L2 and L3) with a long-pass filter (cut-on wavelength of 1,300 nm) to exclude the pump beam. Besides, the lasing beam is split and recombined as a Mach–Zehnder interferometer for self-interference by using beam splitters and gold-coated mirrors (M1 and M2). The optical path difference is controlled by the mirrors’ attitude angles. The lasing beam image is captured by a camera for characterizing its real-space pattern, polarization and self-interference fringes to identify the expected vortex features. The emission spectrum is also detected by a monochromator in the range from 1,540 nm to 1,570 nm with a resolution of ~0.08 nm (see Methods for more details).

## Observation of chiral lasing

**Fig. 5:**
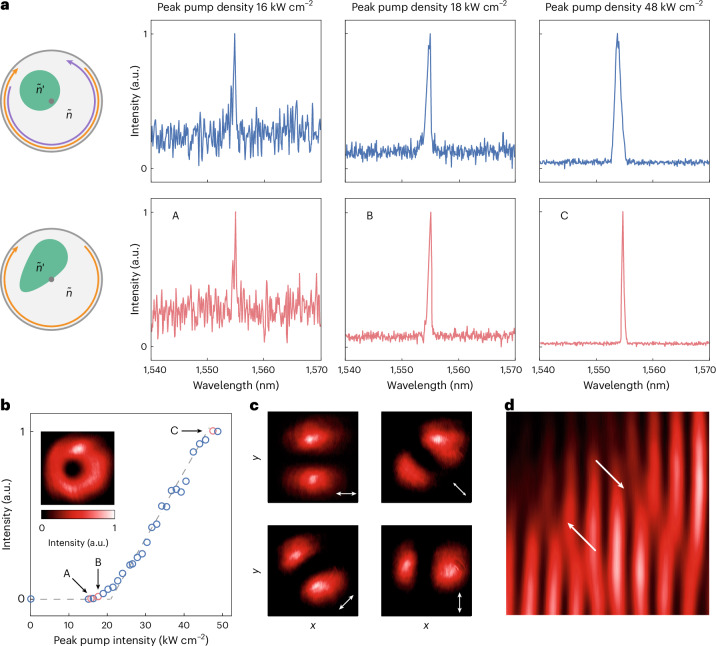
Characterization of lasing oscillation with chiral emission. **a**, The establishing process of lasing oscillation by increasing the pump powers from 16 kW cm^−^^2^ to 48 kW cm^−^^2^, for symmetric circular (upper panel) and asymmetric non-even elliptical (lower panel) pump beams, respectively. Three points from A to C are marked, showing the status from spontaneous emission, threshold lasing, to single-mode lasing. **b**, The power curve of the lasing process is measured, indicating a lasing threshold of 18 kW cm^−^^2^. Inset: a donut shape of the lasing beam in real space. **c**, The polarization-resolved distribution of the vortex beam along 0°, 45°, 90° and 135°, respectively. **d**, The off-centre self-interference pattern of the vortex beam is observed, showing two reversely oriented forks (marked by white arrows) located at the phase singularities as a distinct feature of the spiral phase front. Source data

We first apply a symmetric pump beam with a circular spot of diameter ~5 μm (upper panel, Fig. 5a). At a low pump power of 16 kW cm^−^^2^, spontaneous emission is notable with two small peaks near 1,555 nm, corresponding to the CW and CCW modes that compete in lasing. By increasing the pump power to 18 kW cm^−^^2^, lasing is observed, characterized by the distinct peaks amidst the strongly suppressed spontaneous emission spectrum. Note that the CW and CCW modes coexist in the lasing spectrum, separated by 1–2 nm. The two peaks show different but comparable magnitudes, indicating a lack of dominance in mode competition. When further elevating the pump power to 48 kW cm^−^^2^, the emission becomes stronger while two lasing peaks persist, suggesting that the lasing is not single mode.

The observation aligns with our theory that fabrication imperfection is not adequate for one mode prevailing the competition. Consequently, we opt for asymmetric pumping with a non-even elliptical spot to deliberately break the chiral symmetry, which differentiates the *Q*s of $\left\vert {\psi }_{\mathrm{CW};\,\mathrm{CCW}}^{{\prime} }\right\rangle$. The process of establishing lasing is similar to symmetric pumping when the pump power increases from 16 kW cm^−^^2^ to 48 kW cm^−^^2^ (lower panel, Fig. 5a). Below the lasing threshold, we still found two peaks residing in the spontaneous emission background. Differently, only one peak survives when exceeding the threshold, providing evidence for single-mode lasing.

Further, we measure the lasing power curve (Fig. 5b), in which three data points (labelled A to C) correspond to the spectra illustrated in the lower panel of Fig. 5a. A low lasing threshold of 18 kW cm^−^^2^ is identified attributed to the protection of quasi-BICs. The inset in Fig. 5b shows the lasing pattern in real space, showing an azimuthally uniform donut shape as predicted. We further insert a linear polarizer before the camera to characterize the lasing beam’s polarization along 0°, 45°, 90° and 135° directions. The pattern manifests identical densities along arbitrary directions (Fig. 5c), confirming the rotational invariability of the vortex beam. We notice that the CGR is different from whispering gallery modes, as its dominant polarization aligns along the azimuth rather than the radial direction. Also, besides the lasing mode of (1, 1), high-order CGRs are also observed but less favourable for lasing, since their wavelengths and *Q*s are not optimized in purpose. More data and discussion are presented in Supplementary Section 7.

Finally, we validate the lasing beam’s spiral phase through a self-interference technique. We apply asymmetric pumping to select one mode for lasing, and evenly split the lasing beam and overlap them in real space as a Mach–Zehnder interferometer. A pair of fork fringes with opposite orientations is observed as a distinct feature of phase singularity. The forks’ dislocation points align with the lasing beam centre, at which the fringe separates into two individual branches, confirming that the vortex beam carries ∣*m*∣ = 1. Notably, the forks’ orientation signifies the chirality, which can be either CW or CCW, depending on how chiral symmetry is broken. As a result, we ascertain that the result in Fig. 5d represents CW mode lasing. We also observe CCW mode lasing in another sample, identified by a flipped fork orientation (Supplementary Fig. 10d). It is noteworthy that our lasing beam simultaneously carries a spiral phase vortex from the CGR and polarization winding from the BIC, which behave differently in self-interference. To recognize the observed fork stem from phase singularity, a polarizer before interference is necessary (see Supplementary Section 9 for details).

## Conclusion

In summary, we have reported an observation of chiral emission enabled by collective oscillations of GRs. Leveraging circular boundaries’ scattering, a succession of GRs collectively oscillate in rotational motion, creating a spiral phase front in out-of-plane radiation. The twofold degeneracy of CW and CCW modes was lifted by non-Hermitian coupling effects. We achieve single-mode lasing at a threshold of 18 kW cm^−^^2^ by using asymmetric pumping. The lasing beam’s chiral vortex nature is characterized by polarization-resolved imaging and self-interference fringes.

Collective modes are ubiquitous in photonic^1–3^, plasmonic^4–6^ and quantum wave systems^7,8^, which refer to a cluster of individual modes collectively oscillating as a whole but showing distinctive differences in their field distribution and *Q*s than the single ones. The key to creating collective modes is inducing couplings to connect individual modes. Although most reported collective modes are realized upon tight-binding resonances in real space to enable exotic behaviour in momentum space^50,51^, we have demonstrated that a similar philosophy also holds for coupling momentum-space localized modes (for instance, the GRs) to generate unique real-space distributions according to Fourier duality. We anticipate that, beyond boundary scatterings, other mechanisms such as random disorders, defects and superlattices can also contribute to the couplings even in non-Abelian manners, thus leading to rich and unexplored phenomena of collective oscillation in many-body systems, showing as a direct example in photonic to reflect the famous remarks of ‘more is different’.

CGRs could be a promising way for on-chip vortex generation towards practical applications. Compared with microring vortex lasers^30–34^, CGRs’ surface-emitting nature^52–56^ can facilitate high output power, which is crucial for optical manipulation, detection and communication. Besides, the CGR architecture is compatible with electrical pumping by incorporating asymmetric contacts^57–59^, offering an important stride towards practical lasers. The successes of PhC surface-emitting lasers reveal that the vertical symmetry requirement can be relaxed if the GRs have enough high *Q*s for lasing, thus making the CGR microlaser feasible in a standard semiconductor laser process.

## Methods

### Numerical simulations

The numerical simulation is based on the finite-element method (COMSOL Multiphysics). The band structures in Fig. 1b and the quality factors of the TE-A band in Supplementary Fig. 3a,b are calculated in the 3D unit-cell structures with Floquet periodic boundaries on the sidewalls and perfectly matched layers on both top and bottom. Figure 2 is performed based on 3D simulations of our microlaser design and the direct results obtained by simulations are twofold degenerate eigenmodes independent of time. We remix the twofold degenerate eigenmodes with a ratio of $1:{e}^{\pm i\frac{\pi }{2}}$ to generate CW or CCW modes in Fig. 2b. Although different remixing ratios are also permitted theoretically, these cases can be regarded as mode splitting owing to asymmetric pumping (more details are presented in Supplementary Section 5). Figure 3 and Supplementary Fig. 9 are performed based on 2D simulations of our design, and the CW or CCW modes are directly obtained from the simulations.

### Sample fabrication

The vortex microlaser with undercut PhC structure is designed on an InP-based epi-wafer. As shown in Supplementary Fig. 1a, the active region consisting of six 7.5 nm compressively strained InGaAsP quantum wells and seven 12 nm lattice-matched InGaAsP barriers is sandwiched by undoped 246.5 nm InGaAsP cladding layers. An undoped 1,500 nm InP is used as a wet-etching layer for the fabrication of undercut structure, leading to *z*-direction symmetry for the whole PhC. An undoped 100 nm InGaAsP underneath the etching layer is designed as the etching stop layer. The PhC structures locate at the top most of the epi-wafer. A circular central region with a radius of 10 × *a* (periodicity *a* = 537 nm and radius *r*_A_ = 164 nm) is surrounded by a 50 × 50 array of square-lattice circular holes (periodicity *a* = 537 nm and radius *r*_B_ = 154 nm). The PhC structures are patterned by electron beam lithography and inductively coupled plasma in Cl_2_ at 240 °C. The 1:1 = HCl:H_2_O solution is used to etch InP for the undercut structure under a wet-etching rate of 700 nm min^−1^ at room temperature.

### Measurement and data processing

We use a 1,064 nm pulsed laser with a repetition frequency of 10 kHz and a pulse duration of 2 ns, to pump our vortex microlaser at room temperature. As shown in Fig. 4d, a ×50 objective lens (IOPAMI137150X-NIR, Mitutoyo) is used to converge the pump light to a spot with a diameter of ~5 μm and also collect the vortex beam generated from the sample. The following lens L1 (*f* = 200 mm) confocal with the objective lens, as well as L2 (*f* = 50 mm) and L3 (*f* = 300 mm) forming a 4*f* system, enlarges the real-space image of samples by 300 times in total to generate more in-plane optical path difference in the self-interference experiment. The real-space images are captured by an InGaAs infrared 1,280 × 1,024 CMOS camera (IMX990, Sony). Each interference fringe period in rows possesses ~40 pixels for spatial resolution in Fig. 5d and Supplementary Fig. 10d. Besides, the images, after deducting CMOS intrinsic noise and ambient background noise, are applied data-smoothing along columns with a window size of 30 pixels. Meanwhile, real-space images are also focused into a monochromator with a 600 g mm^−1^ optical grating and an infrared array detector (IsoPlane SCT320 and NIRvana 640, Princeton Instruments), which has a spectral resolution of ~0.08 nm. Polarizer rotation is controlled manually with a maximum uncertainty of ±1° in Fig. 5c and Supplementary Fig. 10c.

### Reporting summary

Further information on research design is available in the Nature Portfolio Reporting Summary linked to this article.

## Online content

Any methods, additional references, Nature Portfolio reporting summaries, source data, extended data, supplementary information, acknowledgements, peer review information; details of author contributions and competing interests; and statements of data and code availability are available at 10.1038/s41565-025-01964-7.

## Supplementary information

Supplementary Information Supplementary Sections 1–10 and Figs. 1–14.

Reporting Summary

## Source data

**Source Data Fig. 1**

**Source Data Fig. 3**

**Source Data Fig. 5**

## Data Availability

All the data supporting the findings of this study are available in the main paper and its Supplementary Information. Additional information can be obtained from the corresponding authors upon request. Source data are provided with this paper.
