# Supplementary material for: Observation of chiral emission enabled by collective guided resonances
Source: Nat Nanotechnol. 2025 Jul 1;20(9):1205–12. doi: 10.1038/s41565-025-01964-7 (PMC12443609; doi:10.1038/s41565-025-01964-7)
Supplement: Supplementary file 1 — Supplementary Sections 1–10 and Figs. 1–14. [file 41565_2025_1964_MOESM1_ESM.pdf]

nature nanotechnology

Supplementary information https://doi.org/10.1038/s41565-025-01964-7

Observation of chiral emission enabled by collective guided resonances

In the format provided by the authors and unedited

Contents

1 Detailed design of chiral lasing of CGRs 2

2 Analysis of omnidirectional mixing of bulk GRs 5

3 Analytical solutions of collective modes within circular boundary PhCs 7

4 Optimization for low-threshold lasing 11

5 Mode Coupling between CW and CCW collective modes 13

6 Discussion of chirality on asymmetric parameters 19

7 Discussion and observation of high-order CGR modes 22

8 Experimental observation of CCW lasing with opposite chirality 24

9 Self-interference patterns of phase vortex, polarization vortex, and their combination 29

10 Statistical and robustness analysis of chiral emission 33

1 Detailed design of chiral lasing of CGRs

We design our structure on an InP-based epi-wafer. As shown in Fig. S1a, the active region is sandwiched by upper and lower InGaAsP cladding layers. The undoped epi layers are grown on n-type substrate. During metal-organic chemical vapor deposition (MOCVD) processes, the buffer layer (130 nm InP), stop layer (100 nm InGaAsP), undercut layer (1500 nm InP), lower cladding layer (246.5 nm InGaAsP), the active region (six 7.5 nm, 8.5% compressively strained wells and seven lattice-matched, 12 nm, −0.3% tensile strained barriers), and upper cladding layer (246.5 nm InGaAsP) are grown from bottom to top in sequence. The photonic crystals (PhCs) are patterned at the top of the epi-layers, and they are designed as a $N \times N$ array of square-latticed circular air holes, where air holes with a slightly larger radius are located in the central circular region to form a heterogeneous PhC. The peripheral PhCs offer a circular boundary of the PBG to confine the light in the lateral direction, and more importantly, give rise to additional momentum in the azimuthal direction.

As a specific example implemented in this work, we design a 50×50 array of air holes (periodicity $a = 537$ nm) with the central region A of radius $R = 10a$ (Fig. S1b). Since air holes must reside at the discrete positions of the PhC lattice, we have to carefully treat the ones near the outer edge of the region A to ensure the roundness of the circular boundary. Specifically, we denote the center positions of air holes as (x, y). For air holes with $\sqrt{x^2 + y^2} \leqslant R + r_A$, we assign a radius of $r_A = 164$ nm to them; otherwise, we let the radius be a different value of $r_B = 154$ nm. Consequently, light traveling in the region A would be reflected by the region B since the TE-A band of the region A is embedded in the band gap between the TE-A and TE-B bands of the region B, as shown in Fig. S1c.

However, the reflection at the hetero-interface is accompanied by energy losses, such as lateral leakage, radiation loss, and scattering loss. Lateral leakage occurs when the geometrical thickness of the boundary region is insufficient to prevent light from traveling through. Since light evanescently travels in the region B, it requires that the geometrical thickness be longer than the penetration depth of the evanescent wave, which is determined by the energy gap of Δ. In our work, we optimized the geometrical thickness

of the region B as $15a$, for which the numerical simulation confirms that the quality factors (Qs) no longer increase as the boundary thickness increases, implying that the lateral leakage is no longer the main cause of energy dissipation in this case.

On the other hand, energy dissipation can also come from the radiation since our PhC cavity operates inside the continuum for both region A and region B, including surface radiation and boundary scattering. By fine-tuning the lattice periodicity while keeping the resonance wavelength nearly fixed at $\sim$ 1550nm, the region A operates at the collective modes of quasi-BICs to suppress the surface radiation (see more details in Suppl. Section 4). At the same time, the region B also has similar performances in terms of radiation strength as the region A, because the two regions share almost identical averaged effective permittivity and vertical profiles (see more details in Suppl. Section 4). Therefore, we conclude that the scatterings at the boundary are the main cause of energy losses in our design due to the mismatch of light momentum between the regions A and B. To realize momentum matching, we optimize the hole radius of the region B to fine-tune the position of the TE-A band in the band gap, which minimizes scattering losses. Furthermore, a larger radius R of the central region A can guarantee a better roundness required by the omnidirectional mixing of quasi-BICs in momentum space. We estimate that $R = 10a$ is sufficient in our design.

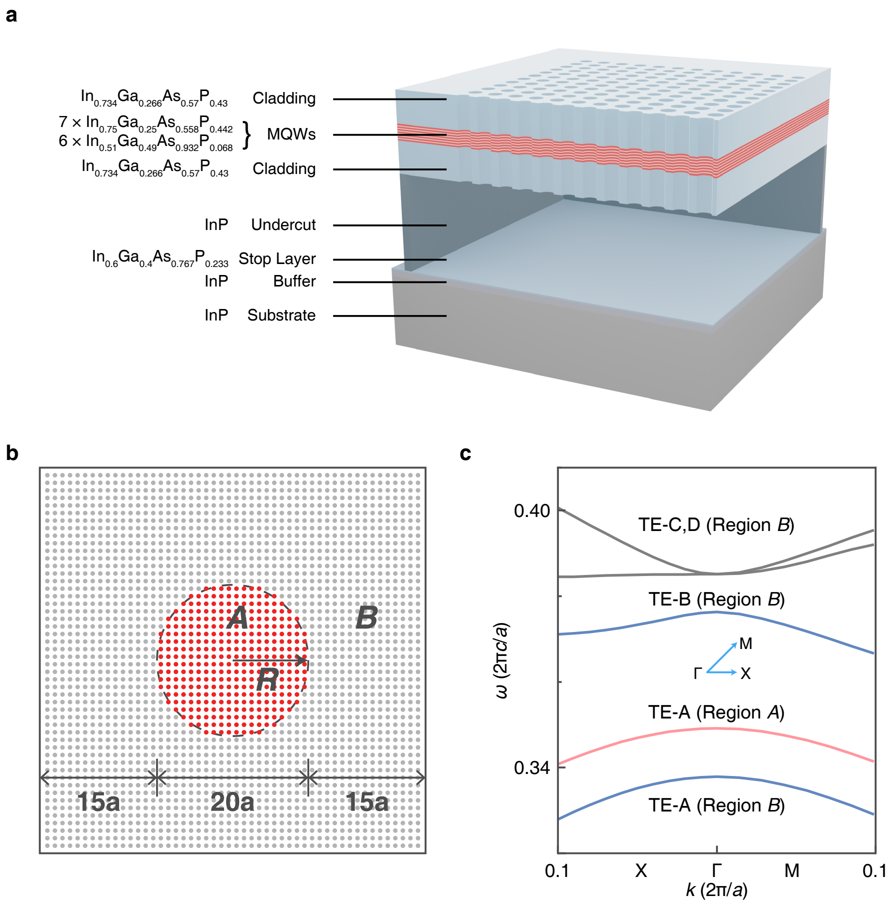

Figure S1: **The detailed structural design and band diagram** (a) The details of the epilayers. The wafer is grown on the InP-based substrate with an undercut layer between PhC and substrate to restore mirror symmetry in z-direction. (b) The total PhC region has a 50×50 array of square latticed circular holes, which consists of the central region A (red, periodicity $a = 537$ nm, hole radius $r_A = 164$ nm, and the region A's radius of $R = 10a$) and the surrounding region B (gray, periodicity $a = 537$ nm, hole radius $r_B = 154$ nm). (c) Band diagram of the designed PhC, where the TE-A band of the region A (red line) are embedded between TE-A and TE-B bands of the region B.

2 Analysis of omnidirectional mixing of bulk GRs

In this section, we discuss how the omnidirectional mixing of GRs creates collective modes in the form of Bessel functions. We start from considering guided resonances in bulk PhC denoted as $|k\rangle$, which has a discrete and determined wavevector due to the periodicity of PhC. Due to boundary scattering (also could be other reasons such as defect scattering, superlattice, disorder, etc.), a set of $|k\rangle$ mixes as a whole in the form of $|\psi\rangle = \sum_j a_j |k_j\rangle$, which is the explicit definition of CGRs.

For the CGRs in a round cavity, recalling the fact to guarantee harmonic stability, the bulk GRs that participated in collective mode hybridization have to align with the iso-frequency contour in momentum space. We then sample the iso-contour at an equal fraction, denoted as a linear space K in the basis $\left\{|k_1\rangle, \cdots, |k_j\rangle, \cdots, |k_N\rangle\right\}$, where the subscript $(1, \cdots, N)$ marks the discrete sampling of the iso-frequency contour. When a given $|k_j\rangle$ hits the boundary, it scatters to other bulk GRs $(\cdots |k_{j-2}\rangle, |k_{j-1}\rangle, |k_{j+1}\rangle, |k_{j+2}\rangle \cdots)$ according to a scattering strength distribution $(\cdots\ \zeta_{j-2,j},\ \zeta_{j-1,j},\ \zeta_{j+1,j},\ \zeta_{j+2,j}\ \cdots)$, also known as the point spread function (PSF). In real space, it can be explicitly written the scattering matrix $\hat{\mathbf{S}}$ as:

$$\mathbf{S} = \begin{bmatrix} \ddots & \vdots & \vdots & \vdots & \iddots \\ \cdots & \zeta_{j-1,j-1} & \zeta_{j-1,j} & \zeta_{j-1,j+1} & \cdots \\ \cdots & \zeta_{j,j-1} & \zeta_{j,j} & \zeta_{j,j+1} & \cdots \\ \cdots & \zeta_{j+1,j-1} & \zeta_{j+1,j} & \zeta_{j+1,j+1} & \cdots \\ \iddots & \vdots & \vdots & \vdots & \ddots \end{bmatrix} \tag{S1}$$

To make the collective modes self-consistent, namely the mode should restore itself after integer times of scattering process, thus the following relationship is required:

$$\hat{\mathbf{S}}^{\mathbf{n}} \vec{V} = \vec{V} \tag{S2}$$

in which n is an integer number, showing that $\vec{V} = [a_1 ... a_j ... a_N]^T$ restore itself after n times of scatterings. Therefore, $\hat{\mathbf{S}}$ follows the n-order cyclic group $G = \left\{g_S, g_S^2, \cdots, g_S^n = e\right\}$ as an Abel group, and the representation of g_S satisfies $\mathbf{S} = \exp\left[2\pi i(j-1)/n\right]$, in which $j \in \{1, 2, \cdots, n\}$. Also, the dispersion is isotropic,

so the basis $|k_j\rangle$ follows the rotational symmetry at arbitrary angles, allowing us to sample the isocontour with an arbitrary N. Thus, the eigenvector $\vec{V}$ can be solved as $\exp[i \times f(2\pi j/N)]$, where $f(2\pi j/N)$ refers to the phase change by circulating the isocontour. This means that the CGR modes should be composed of $|k_j\rangle$ with equal modulus but a gradually changing phase $f(2\pi j/N)$ along the index j. When N approaches infinity, the discrete $|k_j\rangle$ becomes a continuous sampling $|k\rangle$.

According to the Fourier duality, we further find that the CGR has a continuous phase gradient in the real space along the azimuthal direction, thus their mode distribution can be solved in the real space by using the polar coordinate combined with boundary and azimuthal periodic conditions, thereby resulting a set of two-fold degenerate modes in the forms of Bessel functions. To check it, we use the integral expression of the m-th Bessel function and rewrite it as a discrete series:

$$J_m(kr) = \frac{1}{2\pi} \int_{-\pi}^{\pi} e^{i(kr \sin\theta - m\theta)} \, d\theta = \frac{1}{N} \sum_{j=-N/2}^{N/2} e^{i(kr \sin \frac{2\pi j}{N} - m \frac{2\pi j}{N})}, \tag{S3}$$

which reads as $\vec{V} = \left[e^{i[kr\sin(-\pi) - m(-\pi)]}, \cdots, e^{i[kr \sin \frac{2\pi j}{N} - m\frac{2\pi j}{N}]}, \cdots, e^{i[kr\sin(\pi) - m(\pi)]}\right]^T$ and the each a_j has the equal weight that naturally meet the requirement of the omnidirectional scatterings and the Eq. S2. According to the uniqueness theorem, the Bessel functions are the only possible solutions of collective modes in real space under omnidirectional scatterings of circular boundary conditions.

The above discussion also reveals rich possibilities of collective modes. If the boundary is arbitrary and (or) the dispersion is not isotropic, we don't have rotational symmetry to realize at the rotationally invariant solution of $\vec{V}$. The collective mode $\vec{V}$ can have other complex and non-trivial solutions with the cyclic group G, showing more sophisticated distributions in real space. On the other hand, here we assume only the bulk GRs residing on the iso-frequency contour can participate in the mode mixing, which is true when the system is quasi-Hermitian. However, when accounting for the finite lifetimes and non-zero line widths of the modes in the spectrum, the GRs near the isocontour could also contribute to mode mixing, particularly when the band dispersion is flat. Therefore, we expect many unexplored and exotic collective modes to be found in the future, to facilitate sophisticated beam manipulation and other applications.

3 Analytical solutions of collective modes within circular boundary PhCs

In this section, we present the derivation of collective modes in PhC slabs with circular boundaries, to depict the modes we found in numerical simulations and experiments. Our formulation is derived from the perspective of the coupled-wave theory (CWT) framework in polar coordinates, which explains the physics origins of the chiral emission in our design.

We consider an infinite periodic PhC slab on a square lattice and start from the Maxwell equation of electrical fields $\nabla \times \nabla \times \vec{E}(\vec{r}) = k_0^2 \varepsilon(\vec{r})\vec{E}(\vec{r})$, where k_0 is the free-space wave number and $\varepsilon(\vec{r})$ is the permittivity distribution. Without loss of generality, we discuss the TE-like bulk GRs $\vec{E} = (E_x, E_y, 0)$. The light waves inside the PhC must obey Bloch's theorem, and hence, the field $\vec{E}$, as well as permittivity $\varepsilon(\vec{r})$, can be expanded as:

$$E_{x,y}(\vec{r}) = \sum_{mn} E_{x,y;mn}(\vec{r}) e^{-im\beta_0 x - in\beta_0 y}, \tag{S4}$$

$$\varepsilon(\vec{r}) = \varepsilon_0(z) + \sum_{m,n\neq 0} \xi_{mn}(z) e^{-im\beta_0 x - in\beta_0 y}, \tag{S5}$$

where $\beta_0 = 2\pi/a$ represents the reciprocal lattice with the lattice constant of a; m and n are arbitrary integers and ε_0 represents the average permittivity. Substituting Eq. S4 - S5 into the Maxwell equations gives:

$$\begin{aligned}(k_0^2\varepsilon_0 + \partial_{zz} - n^2\beta_0^2 - 2in\beta_0\partial_y + \partial_{yy})E_{x,mn} + (in\beta_0\partial_x + im\beta_0\partial_y + mn\beta_0^2 - \partial_{xy})E_{y,mn} \\ = -k_0^2 \sum_{m'\neq m, n'\neq n} \xi_{m'-m,n'-n} E_{x,m'n'},\end{aligned} \tag{S6}$$

$$\begin{aligned}(k_0^2\varepsilon_0 + \partial_{zz} - m^2\beta_0^2 - 2im\beta_0\partial_x + \partial_{xx})E_{y,mn} + (in\beta_0\partial_x + im\beta_0\partial_y + mn\beta_0^2 - \partial_{xy})E_{x,mn} \\ = -k_0^2 \sum_{m'\neq m, n'\neq n} \xi_{m'-m,n'-n} E_{y,m'n'}.\end{aligned} \tag{S7}$$

Note that due to the translation invariance of infinite periodic PhC, $E_{x,y;mn}$ are in principle constant. However, $E_{x,y;mn}(\vec{r})$ become functions of the spatial position ($\vec{r}$) if the PhC is finite in transverse size. Unlike in our previous works [*1–6*], here we keep the second-order spatial derivative terms to account for their contributions to the slowly varying envelopes of modes. Next, we still assume that the PhC operates at near

the 2nd-Γ point. Accordingly, the basic waves (i.e. $\sqrt{m^2+n^2}=1$) possess dominant energy and we denote them as $E_{mn}(\vec{r})=A_{mn}(x,y)\Theta_0(z)$, where $A_{mn}(x,y)$ can be expressed as a series of plane waves:

$$A_{mn}(x,y)=\sum_j A_{mn,j}(x,y)=\sum_j a_{mn,j}e^{-i(\vec{k}_{\delta,j}\cdot\vec{\rho}+\varphi_j)} \tag{S8}$$

and $\Theta_0(z)$ is the vertical profile shared by the basic waves determined by the average permittivity $\varepsilon_0(z)$ of the slab. For $A_{mn,j}(x,y)$, $\Theta_0(z)$ satisfies:

$$\partial_{zz}\Theta_0(z)+\left[k_{0,mn,j}^2\varepsilon_0-k_{\rho,j}^2\right]\Theta_0(z)=0, \tag{S9}$$

where $k_{0,mn,j}$ is the free-space wave number and $k_{\rho,j}$ is the in-plane wave number satisfying $\vec{k}_{\rho,j}=(m_{x,j}\hat{x}+n_{y,j}\hat{y})\beta_0$. Then, we readily write the field components in Eq. S6 - S7 in terms of Fourier series:

for basic waves:

$$E_{x,mn}=\left[\sum_j -\frac{n_{y,j}}{\sqrt{m_{x,j}^2+n_{y,j}^2}}a_{mn,j}e^{-i(\vec{k}_{\delta,j}\cdot\vec{\rho}+\varphi_j)}\right]\Theta_0(z), \tag{S10}$$

$$E_{y,mn}=\left[\sum_j \frac{m_{x,j}}{\sqrt{m_{x,j}^2+n_{y,j}^2}}a_{mn,j}e^{-i(\vec{k}_{\delta,j}\cdot\vec{\rho}+\varphi_j)}\right]\Theta_0(z); \tag{S11}$$

for other waves:

$$E_{x,m'n'}=\sum_j \lambda_{x,m'n'j}(x,y,z)e^{-i(\vec{k}_{\delta,j}\cdot\vec{\rho}+\varphi_j)}, \tag{S12}$$

$$E_{y,m'n'}=\sum_j \lambda_{y,m'n'j}(x,y,z)e^{-i(\vec{k}_{\delta,j}\cdot\vec{\rho}+\varphi_j)}. \tag{S13}$$

By substituting Eq. S10 - S13 in Eq. S6 - S7 and applying a proper linear combination, that is, $n_{y,j}\times$ (S6) $-m_{x,j}\times$ (S7), we obtain:

$$\begin{aligned}
&(k_0^2\varepsilon_0+\partial_{zz}-n^2\beta_0^2-2in\beta_0\partial_y+\partial_{yy})\times(-n_{y,j}^2A_{mn,j}\Theta_0)\\
&\quad+(k_0^2\varepsilon_0+\partial_{zz}-m^2\beta_0^2-2im\beta_0\partial_x+\partial_{xx})\times(-m_{x,j}^2A_{mn,j}\Theta_0)\\
&\quad+(in\beta_0\partial_x+im\beta_0\partial_y+mn\beta_0^2-\partial_{xy})\times(2m_{x,j}n_{y,j}A_{mn,j}\Theta_0)\\
&\qquad=-\sqrt{m_{x,j}^2+n_{y,j}^2}k_0^2\sum_{m'\neq m,n'\neq n}\xi_{m'-m,n'-n}(n_{y,j}\lambda_{x,m'n'j}-m_{x,j}\lambda_{y,m'n'j})e^{-i(\vec{k}_{\delta,j}\cdot\vec{\rho}+\varphi_j)}.
\end{aligned} \tag{S14}$$

We further simplify the left-hand side of Eq. S14 as:

$$L.H.S. = -\,(k_0^2\varepsilon_0 + \partial_{zz})(m_{x,j}^2 + n_{y,j}^2)A_{mn,j}\Theta_0 + (m^2m_{x,j}^2 + n^2n_{y,j}^2 + 2mm_{x,j}nn_{y,j})\beta_0^2 A_{mn,j}\Theta_0$$

$$+\,2i(m_{x,j}^2 m\partial_x + m_{x,j}n_{y,j}n\partial_x + m_{x,j}n_{y,j}m\partial_y + n_{y,j}^2 n\partial_y)\beta_0 A_{mn,j}\Theta_0$$

$$-\,(m_{x,j}^2\partial_{xx} + n_{y,j}^2\partial_{yy} + 2m_{x,j}n_{y,j}\partial_{xy})A_{mn,j}\Theta_0$$

$$=\frac{1}{\beta_0^2}\left[-(k_0^2\varepsilon_0 - k_{0,mn,j}^2\varepsilon_0 + k_{\rho,j}^2)k_{\rho,j}^2 + (\vec{k}_{\rho,j}\cdot\vec{k}_{mn})^2 + 2i(\vec{k}_{\rho,j}\cdot\vec{k}_{mn})(\vec{k}_{\rho,j}\cdot\nabla_\rho) - (\vec{k}_{\rho,j}\cdot\nabla_\rho)^2\right]A_{mn,j}\Theta_0$$

$$=\frac{1}{\beta_0^2}\left[-(k_0^2\varepsilon_0 - k_{0,mn,j}^2\varepsilon_0 + k_{\rho,j}^2)k_{\rho,j}^2 + (\vec{k}_{\rho,j}\cdot\vec{k}_{mn} + \vec{k}_{\rho,j}\cdot i\nabla_\rho)^2\right]A_{mn,j}\Theta_0. \tag{S15}$$

By collecting the terms with the factor $e^{-i(\vec{k}_{\delta,j}\cdot\vec{\rho}+\varphi_j)}$, Eq. S14 yields to

$$[(k_0^2\varepsilon_0 - k_{0,mn,j}^2\varepsilon_0 + k_{\rho,j}^2)k_{\rho,j}^2 - (\vec{k}_{\rho,j}\cdot\vec{k}_{mn} + \vec{k}_{\rho,j}\cdot\vec{k}_{\delta,j})^2]a_{mn,j}\Theta_0$$

$$= \beta_0 k_{\rho,j}k_0^2 \sum_{m'\neq m,n'\neq n} \xi_{m'-m,n'-n}(n_{y,j}\lambda_{x,m'n'j} - m_{x,j}\lambda_{y,m'n'j}). \tag{S16}$$

Note that here $k_{\delta,j}$ presents the momentum deviation from the Γ point due to the slow-varying envelope, following $\vec{k}_{\rho,j} = \vec{k}_{mn} + \vec{k}_{\delta,j}$. Finally, we find that the amplitudes $a_{mn,j}$ satisfy:

$$(k_0^2 - k_{0,mn,j}^2)a_{mn,j} = \frac{k_0^2}{h_j}\sum_{m'\neq m,n'\neq n}\xi_{m'-m,n'-n}\left[n_{y,j}\int_{PC}\lambda_{x,m'n'j}\Theta_0^*(z)\,\mathrm{d}z - m_{x,j}\int_{PC}\lambda_{y,m'n'j}\Theta_0^*(z)\,\mathrm{d}z\right], \tag{S17}$$

in which $h_j = k_{\rho,j}\int_{-\infty}^{\infty}\varepsilon_0\Theta_0(z)\Theta_0^*(z)\,\mathrm{d}z$. We denote $\vec{V}_j = \left[a_{10,j}, a_{-10,j}, a_{01,j}, a_{0-1,j}\right]^T$ to present a vector of basic waves and we have $j \in$ iso-frequency-contour. Consequently, Eq. S17 leads to an eigenvalue problem in matrix form:

$$k_0^2\vec{V}_j = \mathbf{C}\vec{V}_j. \tag{S18}$$

According to the envelope function approximation, a Γ point mode with a slow-varying envelope is equivalent to a wave packet composed by a combination of bulk modes at off-Γ momenta. Specifically, for our designed PhC, the TE-A band exhibits quadratic curvature in the vicinity of the Γ point, and hence the off-Γ bulk GRs aligned on an isofrequency contour that yields collective modes.

The values of $k_{\delta,j}$ and $e^{-i\varphi_j}$ are determined by the boundary conditions. For our exemplary circular boundary design, the PBG works as a nearly perfect reflective mirror, giving $\sum_{\sqrt{m^2+n^2}=1} E_{x,mn} = 0\big|_{\rho=R}$ and $\sum_{\sqrt{m^2+n^2}=1} E_{y,mn} = 0\big|_{\rho=R}$. The analytical solutions of collective modes are calculated by using the CWT (Fig. S2), showing that the slow-varying envelopes in the transverse plane are in the forms of Bessel functions, which agree well with the results in Fig. 2 in the main text. Physics interpretation of the omnidirectional mixing is presented in Suppl. Section. 2.

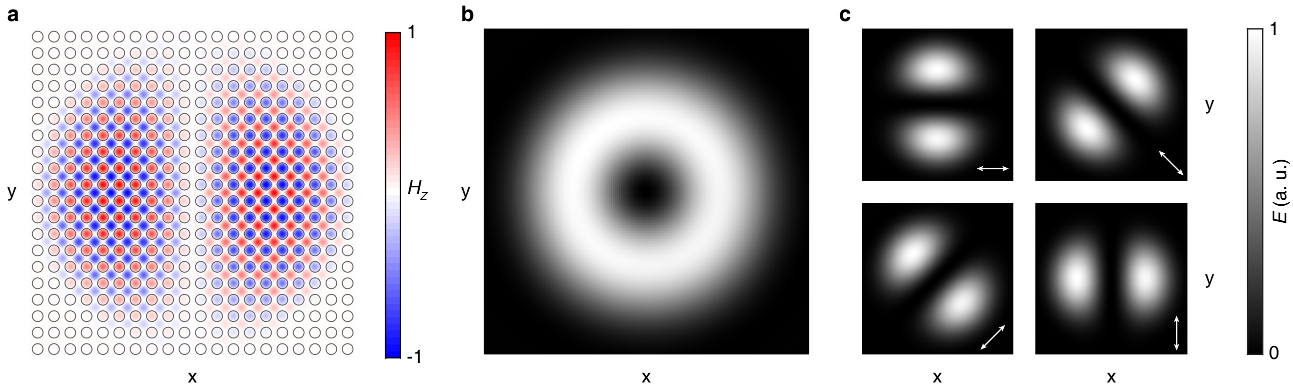

Figure S2: **The collective modes calculated by CWT simulations in polar coordinates** (a) The H_z field distribution of mode $(m, l) = (1, 1)$ at a given snapshot time, corresponds to the simulation by finite element method (FEM) as shown in the lower panel of Fig. 2 (a). (b-c) The distribution of field strength $|E|$ and its polarization-solved patterns at 0°, 45°, 90°, and 135°, which agree with the simulations in FEM and the experimental measurements.

4 Optimization for low-threshold lasing

In this section, we demonstrate that the laser threshold of CGRs can be improved by suppressing their radiation losses by utilizing quasi-BICs [*7, 8*]. Because the collective modes in the circular PhC cavity consist of a series of off-Γ bulk GRs, the radiation can be effectively suppressed by applying the off-Γ BICs at k_{BIC} to match the in-plane momenta $k_{\delta,j}$. The radiation field of the collective modes can be explicitly written as [*1–4, 9*]:

$$E_{x,y;rad} = \sum_j k_0^2 \sum_{\sqrt{m^2+n^2}=1} a_{mn,j}\, \xi_{mn} \left[\int_{PhC} \Theta_0(z') \hat{\mathbf{G}}_\mathbf{0}(z, z', k_{\delta,j})\, \mathrm{d}z' \right] \Bigg|_{z=\pm h/2}, \tag{S19}$$

in which $\hat{\mathbf{G}}_\mathbf{0}$ is the Green's function $\hat{\mathbf{G}}_\mathbf{0} = (\partial_{zz} + \varepsilon_0 k_0^2 - k_{\delta,j}^2)^{-1}$. By tuning the structural parameters of a and r_A, eight ideal off-Γ BICs emerge on the high-symmetric lines along the $\Gamma - X$ and $\Gamma - M$ directions. At the same time, a ring of high values of Q appears at $|\Delta k| = k_{\text{BIC}} = k_{\delta,j}$, in this case, the Qs of the collective modes reach the maximum value.

Specifically, by tuning the structural parameters a and r_A, we found eight off-Γ BICs and one symmetry-protected BIC in the momentum space, carrying integer topological charges on their far-field polarization (Fig. S3a). We calculate the Qs of the TE-A band in the bulk PhC (Fig. S3b), showing that the lifetime diverges to infinity at $k_{\text{BIC}} = 0.034$, giving rise to a ring of high Q values at $|k_{\text{BIC}}| = 0.034$. As we reported previously [*6–8*], the radiations in finite-size PhC can be highly directional, determined by the sizes and shapes of the boundaries. With an isotropic circular boundary, the radiations show equal weights along the contour of Δk_r. They can be effectively suppressed by aligning the in-plane momentum of radiation with the high-Q ring as $\Delta k_r = k_{\text{BIC}}$ (Fig. S3a).

Accordingly, we fine-tune the structural parameters of circular PBG in region B to best suppress the radiation, while keeping the mode wavelength fixed at $\sim$ 1550 nm. As presented in Fig. S3c, we obtain an optimal Q of $\sim 10^6$ at the parameters of $a = 537$ nm, $r_A = 164$ nm, and $r_B = 154$ nm, which is sufficient for low-threshold lasing.

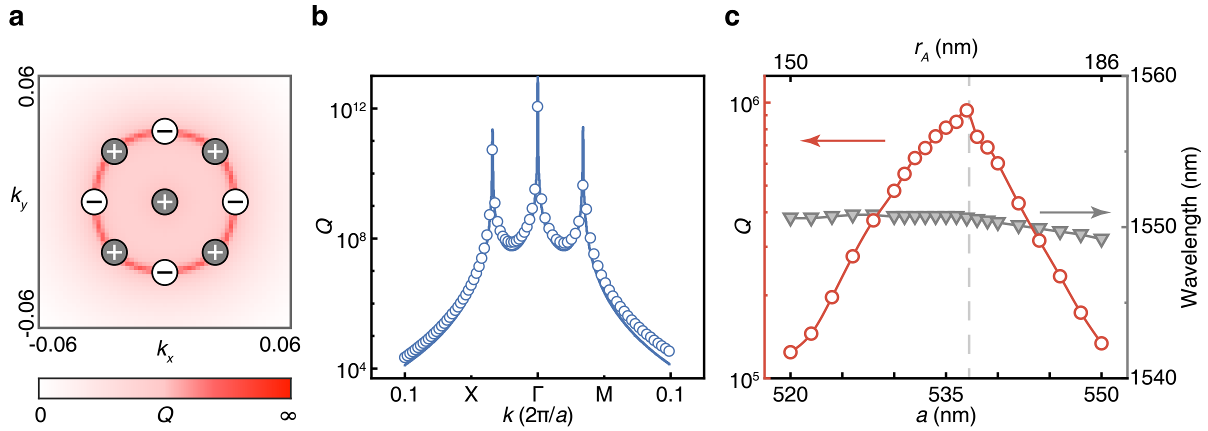

Figure S3: **Optimizing the Qs of CGRs** (a) The Q distribution in momentum space for bulk GRs in the PhC slab, showing eight off-Γ BICs and one symmetry-protected BIC appears near the Brillouin zone center, represented by integer topological charges where the Qs diverge to infinite. (b) The detailed Qs of the TE-A band, the off-Γ BICs are found at $|k_{\text{BIC}}| = 0.034$. (c) The Qs of collective mode (1, 1) in the PhC with circular boundary, the Qs are optimized by tuning structural parameters while keeping the wavelength almost fixed at ∼ 1550, the maximum $Q \sim 1 \times 10^6$ appears at $a = 537$ nm.

5 Mode Coupling between CW and CCW collective modes

We further discuss the coupling of CW and CCW collective modes under asymmetric pump conditions by using a two-mode approximation (TMA) model. The TMA model was previously adopted to deformed micro-disks [*10–13*], as well as micro-rings [*14*], with nano-scatterers to describe the non-Hermitian-induced chirality and the evolution of eigenmodes, successfully demonstrating the coexistence of and differences between two optical cavities with opposite chirality. Using the TMA model, we will show single-mode oscillation behavior when an asymmetric optical pump is applied.

As discussed in the main text and Suppl. Section 2 and 3, the slow-varying envelopes of CGRs are expressed in terms of Bessel functions. For the m-th Bessel function, the spatial distributions of CW and CCW modes are given by:

$$\psi_{CW}(\rho,\varphi) = J_m\left(\frac{\mu_m^l}{R}\rho\right)e^{-im\varphi}, \tag{S20}$$

$$\psi_{CCW}(\rho,\varphi) = J_m\left(\frac{\mu_m^l}{R}\rho\right)e^{im\varphi}, \tag{S21}$$

where R is the radius of region A in Fig. S1b, and μ_m^l denotes the l-th zero of the m-th Bessel function as $J_m(\mu_m^l) = 0$. We investigate the temporal dynamics of the two collective modes by solving a Schrödinger-like equation:

$$i\frac{d}{dt}|\psi\rangle = \mathbf{H}|\psi\rangle, \tag{S22}$$

where the Hamiltonian is a 2×2 matrix and $|\psi\rangle$ is a complex-valued vector linearly combined in the form of $|\psi\rangle = a_{CW}|\psi_{CW}\rangle + a_{CCW}|\psi_{CCW}\rangle$, denoted as $|\psi\rangle = [a_{CW}, a_{CCW}]^T$. Consequently, the beam's chirality can be defined as:

$$\alpha_{ch} = \frac{|a_{CW}|^2 - |a_{CCW}|^2}{|a_{CW}|^2 + |a_{CCW}|^2}. \tag{S23}$$

For a perfectly isotropic cavity, $|\psi_{CW}\rangle$ and $|\psi_{CCW}\rangle$ are two-fold degenerate, and the system's Hamilto-

nian is given by:

$$\mathbf{H_0} = \begin{pmatrix} \Omega_0 & 0 \\ 0 & \Omega_0 \end{pmatrix}. \tag{S24}$$

However, when the fabrication imperfection and optical pump are taken into account, a perturbation term denoted as $\mathbf{\Delta H}$ is introduced, which in general gives a non-Hermitian Hamiltonian of $\mathbf{H} = \mathbf{H_0} + \mathbf{\Delta H}$. Here we focus on the perturbation induced by the asymmetric pump since it is the prominent effect observed in our experiment. Asymmetric pumping introduces non-uniform gain distribution in the sample. Consequently, the pumped area has a complex-valued refractive index $\tilde{n}'$, which differs from the refractive index $\tilde{n}$ of the surrounding areas. To depict the impact of the refractive index difference of $\Delta\tilde{n} = \tilde{n}' - \tilde{n}$, the perturbed Hamiltonian follows:

$$\mathbf{\Delta H} = \begin{pmatrix} \Omega'_{CW} & \kappa \\ \eta\kappa^* & \Omega'_{CCW} \end{pmatrix}, \tag{S25}$$

where Ω'_{CW} (Ω'_{CCW}) represents the frequency perturbation on the CW mode φ_{CW} (CCW mode φ_{CCW}), with their real parts show the frequency shifts and (positive) imaginary parts present the increase of the modal energy. The complex-valued off-diagonal elements κ and $\eta\kappa^*$ describe the asymmetric coupling between CW and CCW modes, and therefore η represents the asymmetric parameter. As a result, the overall effective Hamiltonian can be written as:

$$\mathbf{H} = \mathbf{H_0} + \mathbf{\Delta H} = \begin{pmatrix} \Omega_0 + \Omega'_{CW} & \kappa \\ \eta\kappa^* & \Omega_0 + \Omega'_{CCW} \end{pmatrix}. \tag{S26}$$

The eigenfrequencies and (not normalized) eigenvectors of $\mathbf{H}$ are given by:

$$\Omega_\pm = \Omega_0 + \frac{\Omega'_{CW} + \Omega'_{CCW}}{2} \pm \sqrt{\frac{\left(\Omega'_{CW} - \Omega'_{CCW}\right)^2 + 4\eta\kappa^*\kappa}{4}}, \tag{S27}$$

$$\psi_\pm = \left[2\kappa, (\Omega'_{CW} - \Omega'_{CCW}) \pm \sqrt{\left(\Omega'_{CW} - \Omega'_{CCW}\right)^2 + 4\eta\kappa^*\kappa}\right]^T. \tag{S28}$$

To elucidate the distinctions between symmetric and asymmetric pumping in optical cavities, we first introduce an azimuthal inversion operator $\mathbf{R} : \varphi \mapsto -\varphi$, which transforms azimuthal coordinates from φ to $-\varphi$. We apply $\mathbf{R}$ to the eigenstates $|\psi_{CW}\rangle$ and $|\psi_{CCW}\rangle$ in the perfectly isotropic cavity, as given by Eq. S20 and Eq. S21, and obtain:

$$\mathbf{R}|\psi_{CW}\rangle = |\psi_{CCW}\rangle, \quad \mathbf{R}|\psi_{CCW}\rangle = |\psi_{CW}\rangle. \tag{S29}$$

Since the perfectly isotropic cavity remains unchanged under inversion, the inversion operator $\mathbf{R}$ and the Hamiltonian $\mathbf{H_0}$ commute, i.e.,$[\mathbf{R}, \mathbf{H_0}] = 0$, and they share common eigenstates.

In the case of cavities under the symmetric pump, where the pumped area exhibits even parity relative to a line through the center of the isotropic cavity, as shown in Fig. S4b, we can always refer to this line as the origin of the azimuthal coordinates without loss of generality. This indicates that the perturbed Hamiltonian $\mathbf{\Delta H}$ commutes with $\mathbf{R}$, i.e. $[\mathbf{R}, \mathbf{\Delta H}] = 0$. Consequently, the overall Hamiltonian $\mathbf{H}$ and the inversion operator $\mathbf{R}$ satisfy the commutation relation:

$$[\mathbf{R}, \mathbf{H}] = 0, \tag{S30}$$

and share common eigenstates $|\psi_\pm\rangle$. The eigenvalue of $\mathbf{R}$ is denoted as r, which can be derived by applying $\mathbf{R}$ twice to $|\psi_\pm\rangle$ and noting that the cavity remains unchanged under two consecutive inversion operations:

$$\mathbf{R}\left(\mathbf{R}|\psi_\pm\rangle\right) = r^2|\psi_\pm\rangle, \tag{S31}$$

where r takes the values $r = \pm 1$. Combining Eq. S26–S31, we deduce that Ω'_{CW}, Ω'_{CCW} and η should satisfy $\Omega'_{CW} = \Omega'_{CCW}$ and $\eta = 1$. Therefore, the eigenfrequencies and eigenvectors of $\mathbf{H}$ are simplified as:

$$\Omega_\pm = \Omega_0 + \Omega' \pm |\kappa|, \tag{S32}$$

$$\psi_\pm = [\kappa, \pm|\kappa|]^T, \tag{S33}$$

where Ω' is the common frequency shift of eigenstates due to the symmetric pump. From Eq. S32, we infer that eigenstates in the symmetric perturbed cavity differ only in the real part of their frequency but still remain the same (positive) imaginary parts, thus impeding the single-mode oscillation. Furthermore,

Eq. S33 indicates that the chirality of both two eigenstates is zero: $\alpha_{ch} = 0$. We define the amplitude ratio between CW and CCW modes ($|a_{\mathrm{CW}}| : |a_{\mathrm{CCW}}|$) to represent "chiral purity". As a reference, the self-interference patterns for different chiral purities are shown in Fig. S4d. When $\alpha_{ch} = 0$, the ratio becomes $0.5 : 0.5$ and the "fork pattern", a distinct feature of vortex beams, disappears. We observed the self-interference pattern for the symmetric pump as shown in Fig. S4c, which is consistent with our theoretical prediction in Fig. S4d.

In case of cavities under the asymmetric pump as shown in Fig. S4a, the perturbed Hamiltonian $\mathbf{\Delta H}$ and the inversion operator $\mathbf{R}$ do not commute with each other, resulting in the non-commutation relation between the overall Hamiltonian $\mathbf{H}$ and $\mathbf{R}$, denoted as:

$$[\mathbf{R}, \mathbf{H}] \neq 0. \tag{S34}$$

Note that the two eigenvectors become non-orthogonal when $\eta = 0$ and $\Omega'_{CW} = \Omega'_{CCW}$, indicating that the mode pair is at the so-called exceptional points (EPs) in parameter space, where only one rotating wave, either CW or CCW, exists with chirality $\alpha_{ch} = \pm 1$. In Fig. S5, we show the EPs in 2D parameter space of $Im(\tilde{n}_1)$ and θ denoted in Fig. 3a and found an EP at $Im(\tilde{n}_1) = 0.035$ and $\theta = 100.15°$. Otherwise, when $\eta \neq 0$ and $\eta \neq 1$, the differences in Qs of two eigenstates emerge and can contribute to single-mode oscillation as a hybridization of CW and CCW modes, as shown in Fig. 3b. We present different "chiral purity" in Fig. S4d. We find that the pattern of $|a_{\mathrm{CW}}| : |a_{\mathrm{CCW}}| = 0.65 : 0.35$ with chirality $\alpha_{ch} = 0.55$ is the closest to our experimental results in Fig. 5d.

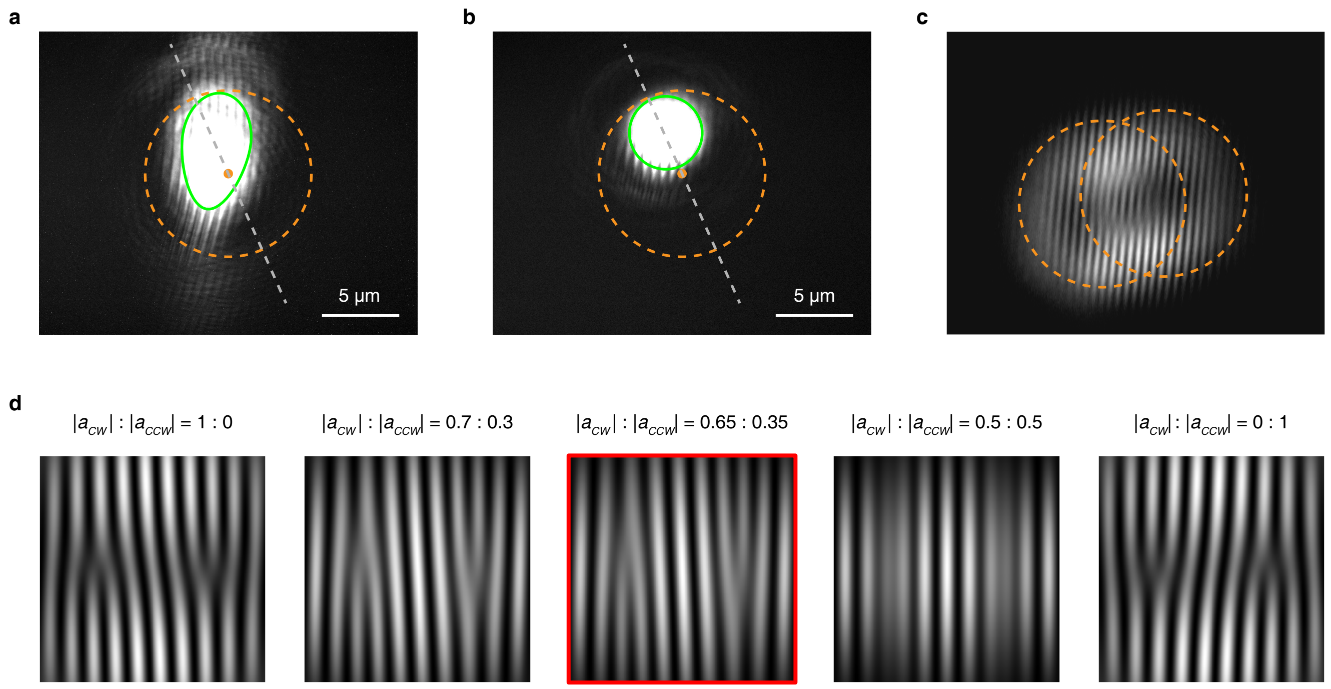

Figure S4: **The principles of coupling between CW and CCW modes** (a) The observed optical cavity under the asymmetric pump with a non-even elliptical pumped area. (b) The observed optical cavity under the symmetric pump with a circular pumped area. (c) Experimental results of self-interference patterns in the case of the symmetric pump. (d) The theoretical self-interference patterns at different mixing ratios of CW and CCW modes. The pattern of $|a_{\mathrm{CW}}| : |a_{\mathrm{CCW}}| = 0.65 : 0.35$ with chirality $\alpha_{ch} = 0.55$ closely matches the experimental results in Fig. 5d.

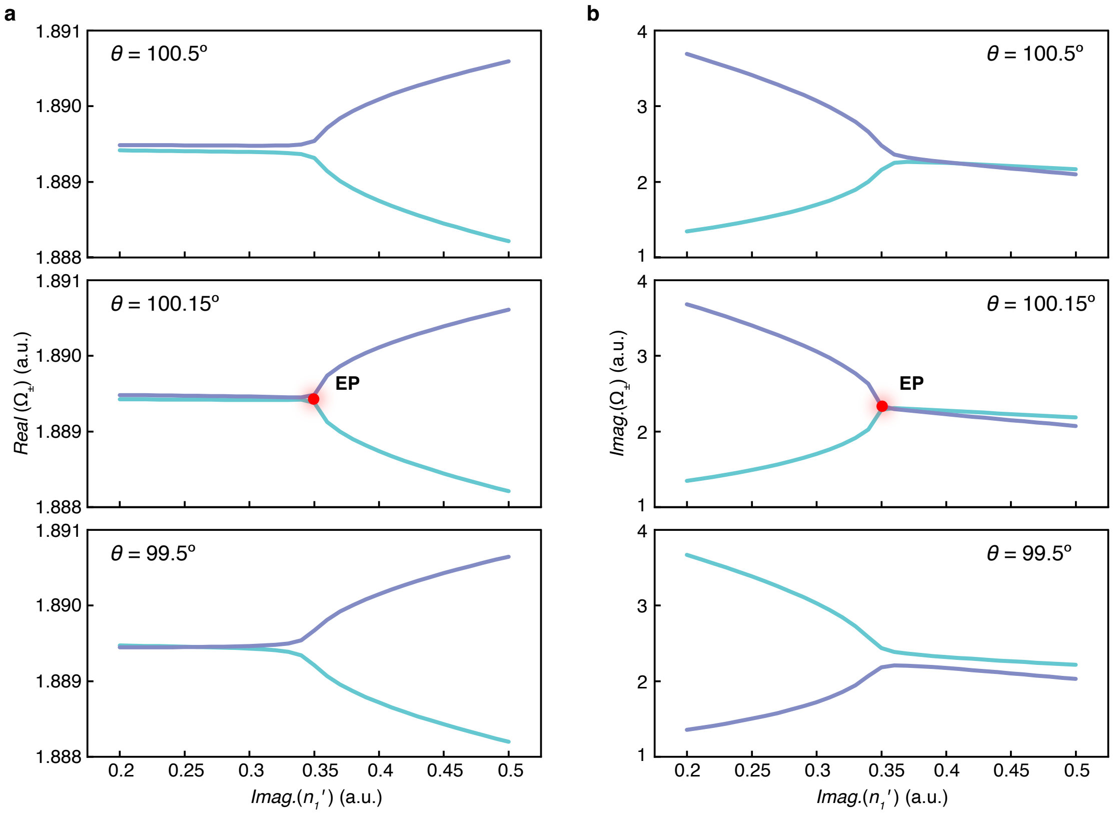

Figure S5: **The eigenfrequency $\Omega_{\pm}$ evolution as a function of $Im(\tilde{n}_1)$ and θ.** $\tilde{n}_1$ denotes the complex index of the pumped area and θ denotes the relative angle of two pump beams, as shown in Fig. 3a. (a) Real part of the eigenfrequency $\Omega_{\pm}$. (b) The imaginary part of the eigenfrequency $\Omega_{\pm}$.

6 Discussion of chirality on asymmetric parameters

In this section, we present some quantitative analysis of asymmetric pumping. To evaluate how asymmetry strengths influence the characteristics of the modes. We first calculated the complex bands and chirality using the eigenvectors solved from Eq. S27 and S28. As shown in Fig. S6, when departing from the EP by increasing the asymmetric parameter η that describes the asymmetric coupling between CW and CCW modes, one mode has a higher Q that is more favorable for lasing. The chirality reaches a maximum value of 1 at the EP and drops when departing from the EP.

In addition, we present simulations to evaluate how the asymmetry strengths influence the characteristics of the modes. Specifically, we keep the total gain area unchanged across the simulations and scan the imaginary refractive index $Im(\tilde{n}_1)$ of the pumped region ($\tilde{n}_1$), together with the relative angle (θ) of two pump spots as a 2D parameter space. The EP resides at $Im(\tilde{n}_1) = 0.035$, $\theta = 100.15°$. The parameter sweeping covers a range of $Im(\tilde{n}_1) = [0.015, 0.06]$ and $\theta = [97°, 125°]$. The calculated complex bands in the parameter space are presented in Fig. S7a and S7b.

We can directly identify the chirality from the mode field distribution. For an example, for $Im(\tilde{n}_1) = 0.015$ and $\theta = 100.15°$, the normalized electric field of eigenmode A_1 is plotted in Fig. S7c, showing as a pair of equal-density lobes aligned with the 45° direction, respectively, rather than a donut shape. Moreover, when changing the phases of the A_1 at different times, its field distribution H_z is almost invariant (Fig. S7d), indicating that the mode no longer rotates with determined chirality.

Using this criterion, we identify which asymmetry strength could ruin the chirality. When $Im(\tilde{n}_1)$ deviates 70% (to A_1 and A_2) or θ changes 19.8% (to B_1 and B_2) from the EP, the mode chirality disappears, indicating the chirality is more sensitive to asymmetric orientation than its strength. The real frequencies splitting at the chiral vanishing points are read as $\sim 0.15\%$ of the central frequency, corresponding to about ~ 2 nm of mode spacing in the spectrum. In our experiment with an asymmetric pump, two peaks separated at $1 \sim 2$ nm are observed from the lasing spectrum (Fig. 5 and Fig. S10), supporting that our experiment has

similar asymmetry strength compared to the simplified model. Therefore, we conclude that the asymmetry of the pump beam should be kept in a range of 10 ~ 20 % to ensure the effectiveness of chiral emission.

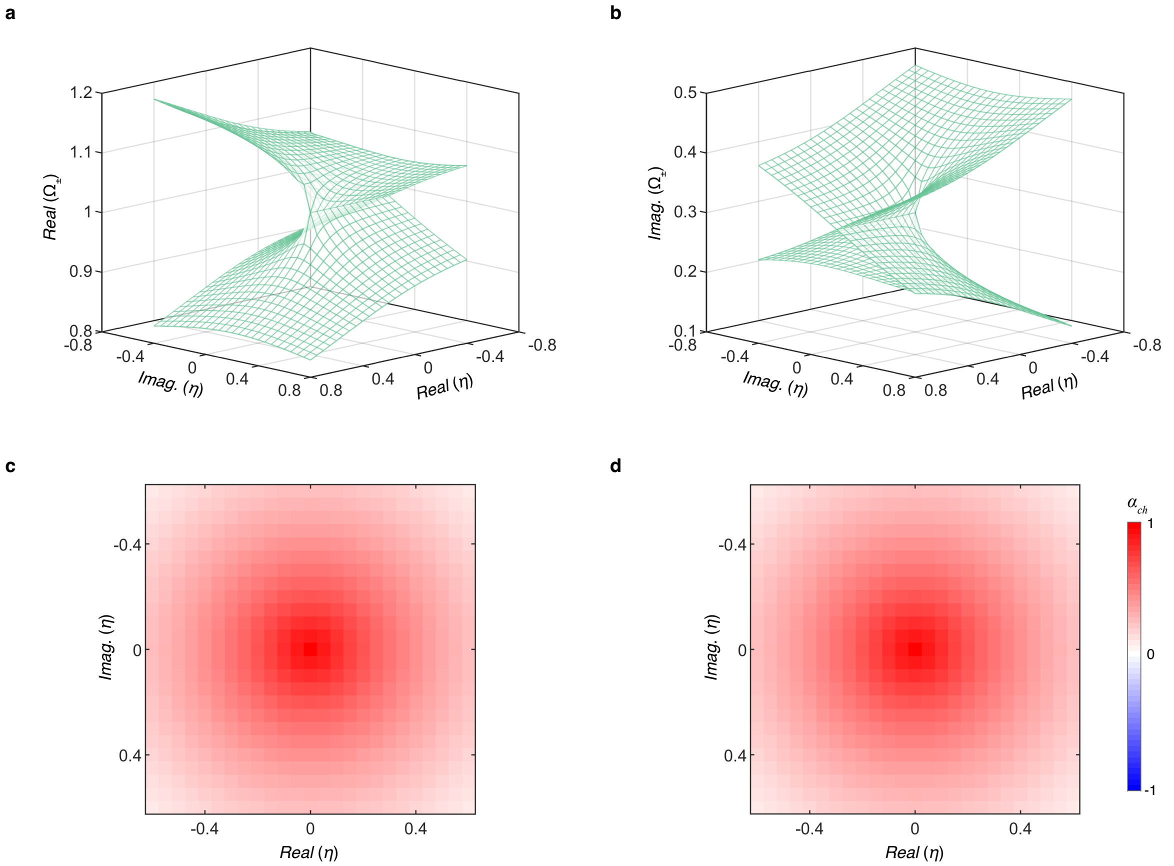

Figure S6: **The complex band and chirality as a function of complex asymmetric coefficient η with** $\Omega_0 = 0.8$, $\Omega'_{CW} = \Omega'_{CCW} = 0.2 + 0.3i$, $\kappa = 0.2 - 0.1i$. (a) The real part of the eigenfrequency $\Omega_\pm$. (b) The imaginary part of the eigenfrequency $\Omega_\pm$. (c) The chirality α_{ch} of the eigenmode $|\psi_+\rangle$. (d) The chirality α_{ch} of the eigenmode $|\psi_-\rangle$.

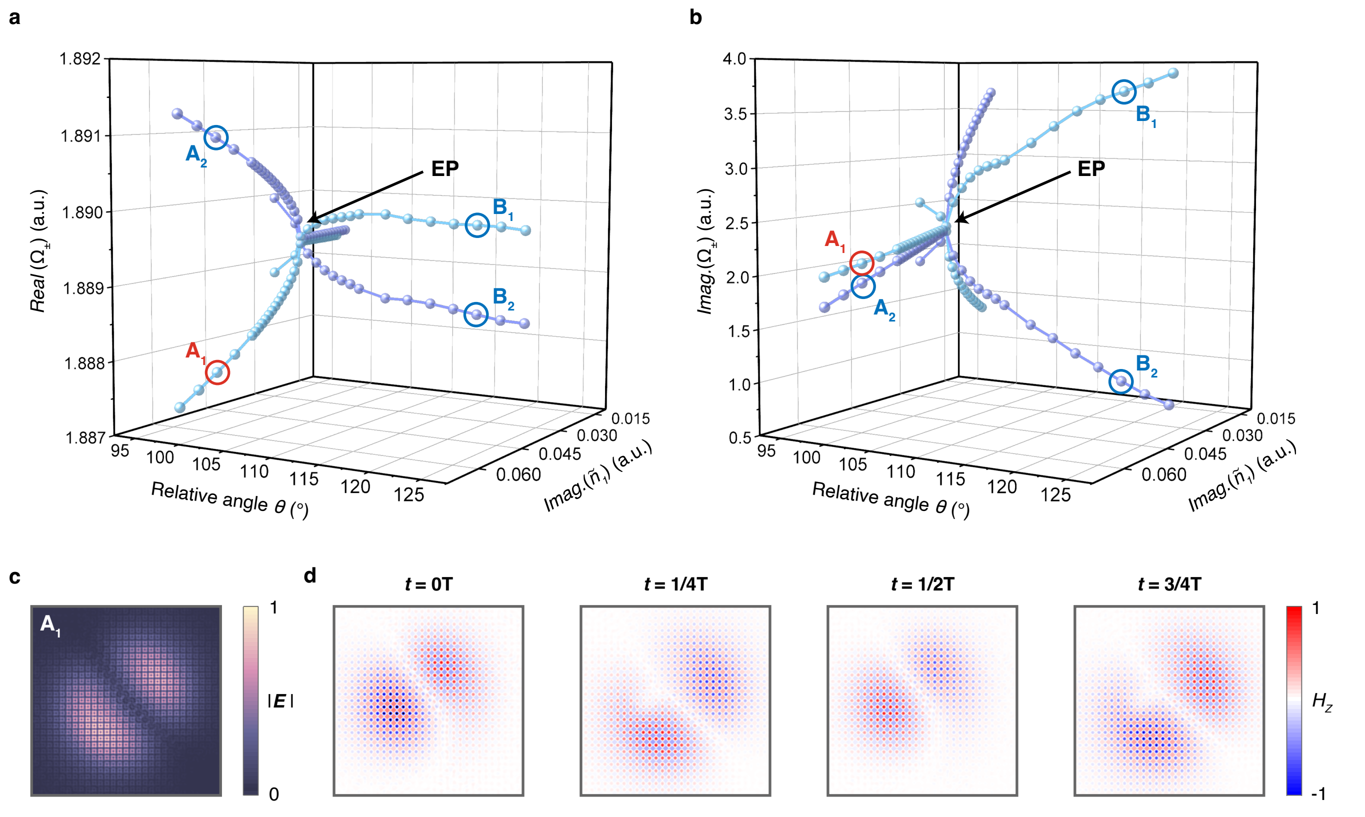

Figure S7: **Numerical results of complex bands and chirality as a function of $Im(\tilde{n}_1)$ and θ.** (a) The real part of the eigenfrequency $\Omega_{\pm}$. (b) The imaginary part of the eigenfrequency $\Omega_{\pm}$. (c) The time-averaged electrical field distribution $|\boldsymbol{E}|$ when the mode chirality disappears. A_1 is at $Im(\tilde{n}_1) = 0.06$, $\theta = 100.15°$. (d) The snapshot magnetic fields H_z of A_1 in a time interval of $1/4T$ indicate the vanishing of rotating. The chirality also disappears at the points A_2, B_1, and B_2. All simulations use COMSOL Multiphysics.

7 Discussion and observation of high-order CGR modes

It is worth emphasizing that the mode $(m, l) = (1, 1)$ is the only lasing mode we observed in the experiment due to our optimized design. However, the theory predicts that there are also other high-order collective modes with different (m, l) in the system. The quantum number m represents how many times the phase folds in the range of $[0, 2\pi)$, which can in principle be any integers other than $m = 1$, representing high-order vortex beams.

Our design has been optimized to lase at the collective mode of $(m, l) = (1, 1)$. We also fabricate the sample with less ideal parameters and pump it with larger power. As a result, it simultaneously lases at multiple wavelengths as shown in the lower panel of Fig. S8, while each peak corresponds to a different order collective mode we predicted in theory. Using the numerical simulation and CWT theory, we identify the peaks and classify them into a series of quantum numbers of (m, l), as shown in the upper panel of Fig. S8. The spectrum sequencing and spacings of individual collective modes match well with the theory and simulation. More importantly, it is found that except for $m = 0$, other collective modes are in two-fold degeneracy because of the co-existence of CW and CCW modes, which further validates our theory.

We are optimistic about designing a particular structure to enable high-order vortex lasing. The selective lasing of the high-order vortex can be achieved by following aspects: first, we can tune the structural parameters (for instance, the lattice constant a) to make the target mode residing at a favorable wavelength in the material gain spectrum for lasing, referred to as "gain selection"; second, we can manipulate the topological charge of radiation through designing structure parameters to best match the targeted order of resonance in promoting its Q value for mode competition, referred to as "Q selection". As a result, it can realize high-order vortex beam beyond the limitation of lattice symmetry, which is one of the major drawbacks of the PB phase approach of vortex beam generation in the momentum space.

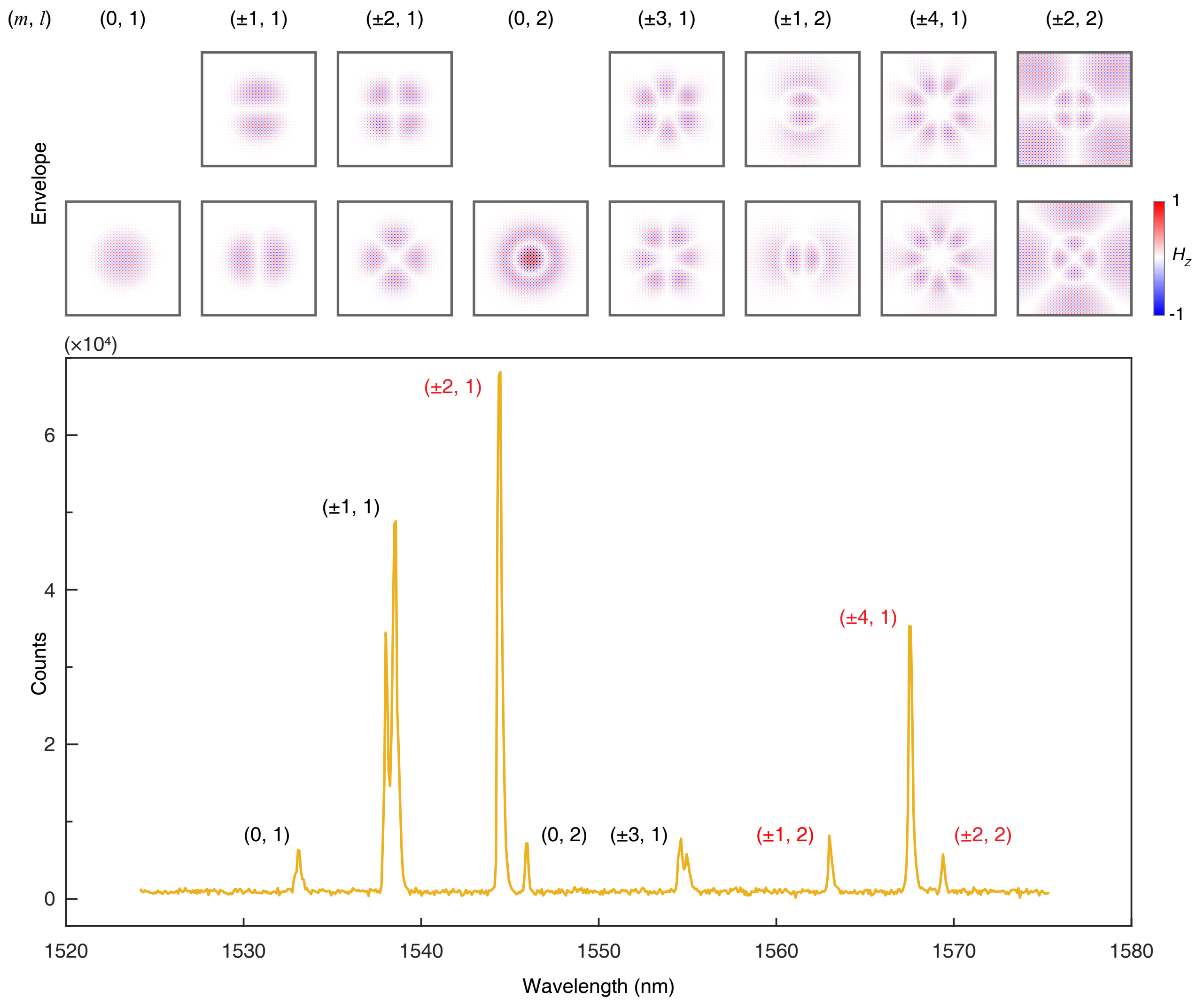

Figure S8: **The high-order collective modes** The H_z field distributions of a series of collective modes with quantum numbers (m, l) (upper panel). The peaks of high-order collective modes are observed in the lasing spectrum when large pump power is applied (lower panel). The modes marked by red do not show evident two-fold degeneracy as the theory predicts; the reason could be their lasing is not quite stable, or the spectrum difference is beyond the solution of the measurement.

8 Experimental observation of CCW lasing with opposite chirality

The theory predicts that a CCW mode also exists in the system whose chirality is opposite to the CW mode which we have elaborated on in the main text. According to the inversion symmetry, we can easily obtain another EP for CCW mode in the parameter space at $Im(\tilde{n}_1) = 0.035, \theta = -100.15°$, which is identified by the snapshots of its donut-shape electrical field strength and CCW rotation motion of magnetic fields (Fig. S9). The experimental results of CCW mode are shown in Fig. S10, showing a different orientation of the fork pattern compared to the results of CW mode we presented in Fig. 5. As mentioned in Suppl. Section 4, the flip of fork orientation is a distinct feature to distinguish CW and CCW collective modes.

Specifically, here the structural parameters of the sample are characterized as the periodicity of a = 537 nm and diameter of r_A = 162 nm. When the sample is under a low pump power of 16 kW/cm^2 with a circular spot (upper panel in Fig. S10a), two small optical resonance peaks develop around the wavelength of 1562 nm, representing the coexistence of CW and CCW modes. Further, we increase the pump power to a slightly higher value of 18 kW/cm^2, showing that the laser oscillation is established. At this point, the peaks of CW and CCW modes still coexist in the laser spectrum with comparable intensities, indicating the presence of mode competition. By increasing the pump power to 47 kW/cm^2, a notable enhancement in emitted power occurs, but we still find the two peaks persist, denying the microlaser operating in single-mode oscillation.

We notice that asymmetric pumping is necessary to excite the CCW mode (as shown in the lower panel of Fig. S10a). In this case, by increasing the pump power from 16 kW/cm^2 to 47 kW/cm^2, the process of lasing oscillation establishment is similar to the case of symmetric pumping mentioned above; however, above the lasing threshold, only one single lasing peak (CCW mode) is evident in the spectrum, indicating that the CW mode is suppressed during mode competition because an appropriate alignment of asymmetric pumping creates a considerable difference in Qs between CW and CCW collective modes, accordingly, the CCW mode prevails in lasing. The curve of pump power versus emission power at room temperature is shown in Fig. S10b, in which the three points from A to C are marked, corresponding to different pump

power densities in Fig. S10a. A low lasing threshold is observed at 18 kW/cm^2 because of the high-Q nature of collective quasi-BICs.

We similarly characterize the vortex features of the CCW mode by using polarization-resolved imaging and self-interference patterns. The real-space pattern is shown in the inset of Fig. S10b, exhibiting a standard donut-shaped configuration that agrees well with the envelope of the Bessel function $(m, l) = (1, 1)$. Fig. S10c shows the polarization distribution of the CCW mode: the donut-shaped pattern becomes two bright lobes orthogonally aligned to the orientation of the linear polarizer.

However, as we emphasized, the donut patterns combined with polarization-resolved distributions are not a sufficient condition to distinguish the CW and CCW modes. The only way to distinguish them is from the orientation of fork patterns in the self-interference observation. Therefore, we similarly split the vortex beam equally into two parts and overlap them by using the Mach-Zehnder interferometer setup, while maintaining the same relative position in real space as in Fig. 4d. Not surprisingly, we observed a pair of fork patterns with opposite orientations, in which the dislocation points precisely aligned at the center of each vortex beam. At the center of the vortex beam, the single fringe separates into two branches, thereby confirming a quantum number of $|m| = 1$. More importantly, we found the forks in Fig. S10d are flipped their orientation relative to the pattern of CW mode presented in Fig. 5d, confirming that the vortex beam in this experiment carries an opposite sign of chirality compared to the result in the main text, so we identify it is a CCW collective mode of $m = -1$.

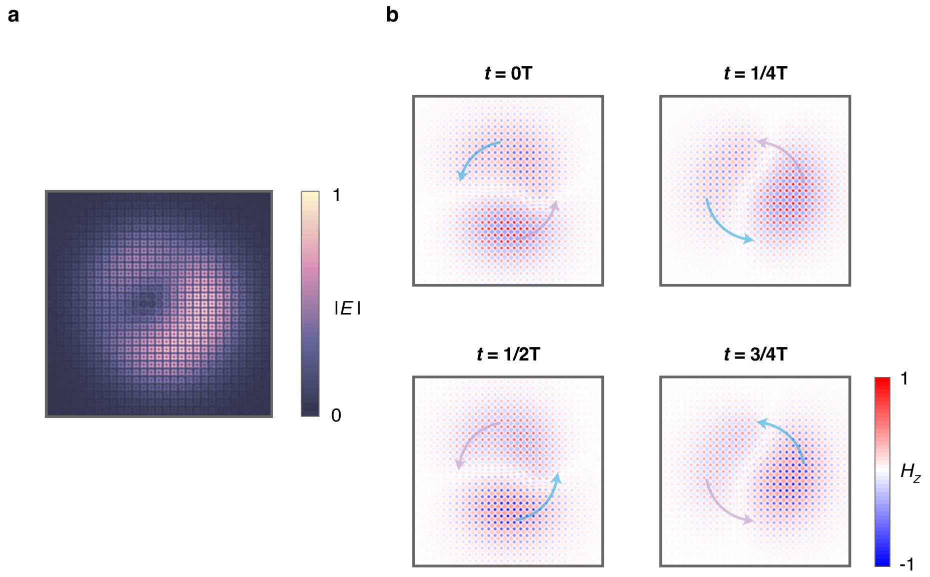

Figure S9: **The CCW mode at the EP** (a)The donut-shape electrical field strength $|\boldsymbol{E}|$ in real space. (b)The evolution of snapshot magnetic field H_z, showing the rotation motion along CCW direction.

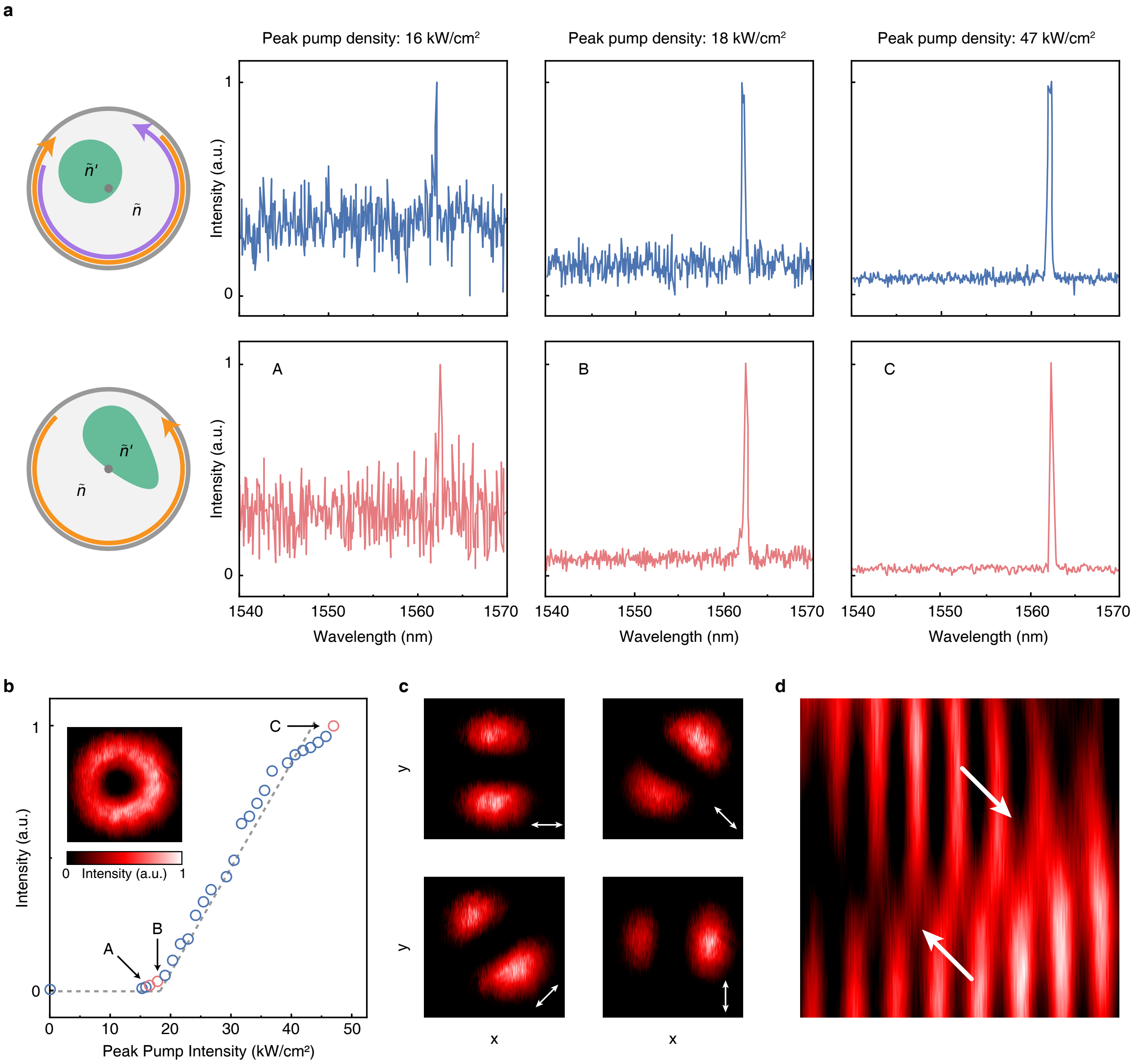

a
Peak pump density: 16 kW/cm²
Peak pump density: 18 kW/cm²
Peak pump density: 47 kW/cm²
Intensity (a.u.)
A
B
C
Wavelength (nm)
b
Intensity (a.u.)
Peak Pump Intensity (kW/cm²)
c
d

Figure S10: **Observation of vortex lasing with opposite chirality** (a) The establishing process of lasing oscillation by increasing the pump powers from 16 kW/cm^2 to 47 kW/cm^2, for symmetric (upper panel) and asymmetric (lower panel) pump conditions, respectively. Three points from A to C are marked, showing the status from spontaneous emission, threshold lasing, to single-mode lasing. (b) The power curve of the vortex microlaser is measured, indicating a low threshold of 18 kW/cm^2. The inset shows a donut shape of the lasing beam in real space. (c) The polarization-resolved distribution of the vortex beam along 0°, 45°, 90°, and 135°, respectively. (d) The off-center self-interference pattern of the vortex beam is observed, showing two reversely oriented forks (marked by white arrows) located at the phase singularities as a distinct feature of a phase vortex beam. Importantly, the fork orientation is flipped compared to the results in Fig. 5 (CW mode), thus we identify here the CCW mode with opposite chirality is lasing.

9 Self-interference patterns of phase vortex, polarization vortex, and their combination

In singularity optics, vortex beams carry winding quantities in their wavefront, which could be phase, polarization, or a combination of them. We clarify that different vortex beams can create distinct features in their self-interference patterns, and we discuss and show several examples in this section.

Phase vortices refer to a type of light beam with a winding phase singularity in the wavefront while keeping their electric field direction, namely polarization invariant. Consequently, phase vortices are also known as scalar vortices that carry OAMs, which can be generated by making a light beam pass through helical phase plates. In contrast, polarization vortices show spatial winding of the electric field direction, featuring a polarization singularity at the center but the wavefront remains equal in phase. Because polarization is a vectorial quantity, such beams are known as vectorial vortices. A well-known example is the BICs in periodic photonic structures, which exhibit a vector polarization vortex in the momentum space. We argue that the phase and polarization winding can be combined, namely during the light propagation, it not only accumulates phase windings in the azimuthal direction but also rotates its polarization, similar to the behavior of the light propagating in a micro-ring; therefore, we refer to it as "ring vortex". The vortex beam proposed in this work also belongs to this type.

The vortex beams mentioned above behave differently in self-interference experiments. Specifically, we consider a light beam split into two equal parts and then overlap them at an off-center position in real space. For the phase vortex beam, due to the phase singularity, a pair of fork patterns with opposite orientations would be observed whose dislocation points align with the center of each vortex beam. As an example, we show the interference pattern of a phase vortex with the quantum number of $m = 1$ in the middle panel of Fig. S11b, where we suppose the phase vortex beam is linearly polarized with the electric field of $(E_x, E_y) = (1, 1)$. We denote $I_{x,y} = |E_{x,y}|^2$ as the intensity of $E_{x,y}$; I denotes the total intensity as $I = I_x + I_y$ since the x, y polarizations are orthogonal.

For a vectorial vortex, the polarization of the light is no longer invariant. As shown in Fig. S11a, we

plot the interference pattern of the beams with the polarization winding number of $q = 1$, and the electric field is normalized as $|\boldsymbol{E}| = 1$. As shown in Fig. S11a, the split beams have opposite polarization in the overlap region between their beam centers, represented by the yellow and blue arrows, respectively. Such a polarization configuration results in an extra phase difference for interference. Therefore, the interference fringes in the center region have a $\pi-$shift compared to those in the outer region, leading to a circle of dislocation points in the total (vectorial) interference pattern of I, as shown in the upper panel of Fig. S11b.

Differently, the ring vortex possesses helical phase and winding polarization in the wavefront at the same time, making the interference pattern similar to a combination of both phase and polarization vortices, as shown in the lower panel of Fig. S11b. While a pair of fork patterns still exists because of phase singularities, the circle of dislocation points with $\pi-$shift fringes intersects the forks and connects the phase singularities. This unique interference pattern of the ring vortex can cause difficulties in fork recognition; therefore, we should avoid the influence of vector vortices to confirm the existence of phase singularities in experiments.

A simple way to get rid of the polarization dislocation points is to place a linear polarizer before the observation, which turns the wave interference from vectorial to scalar. Fig. S12a shows the experimental observation of our vortex beam, which matches well with the theoretical prediction calculated in Fig. S11. Further, we add a polarizer at 30° in front of the Mach-Zehnder interferometer. A pair of forks without circular dislocation points can be found in the interference pattern (Fig. S12b); the enlarged image is shown in Fig. 5d in the main text, thus confirming our observed fork pattern originated from phase vortex but not polarization vortex.

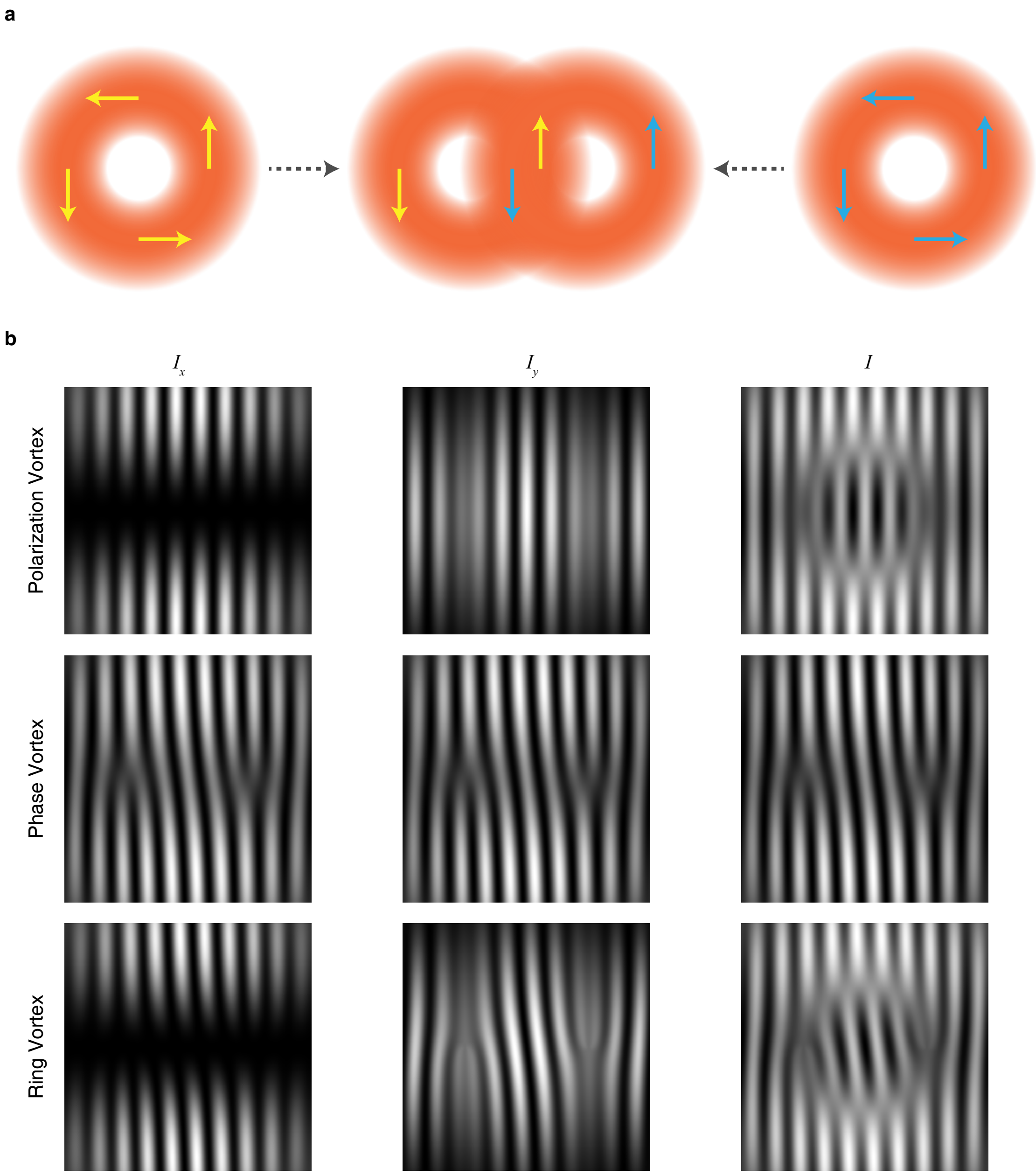

Figure S11: **Self-interference patterns of phase, polarization vortices, and their combination** (a) The principle of interference between two vectorial (polarization) vortices, in which an extra phase difference is induced due to their vectorial nature. (b) The field intensity of E_x, E_y, and total intensity for the polarization vortex (upper panel), phase vortex (mid panel), and their combination as ring vortex (lower panel), respectively.

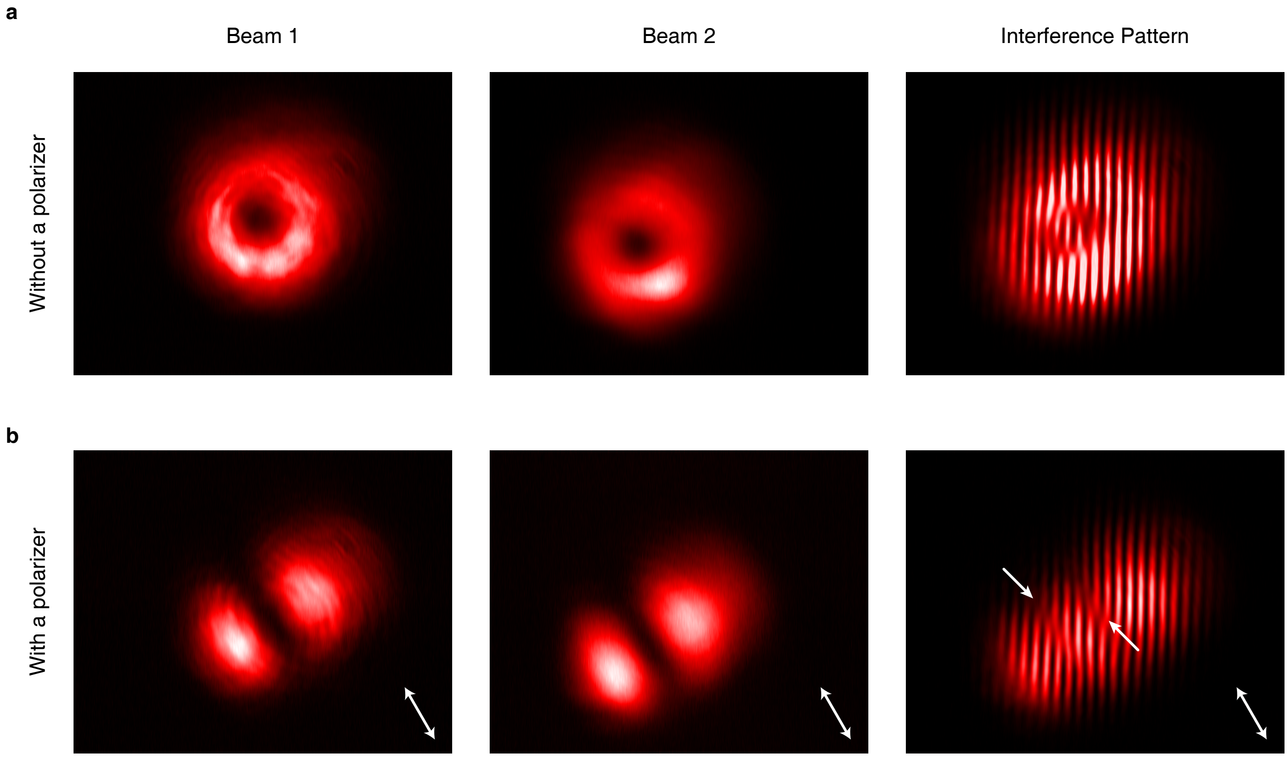

Figure S12: **Self-interference pattern of unpolarized and polarized light** (a) When a polarizer is absent before observing the self-interference pattern in the experiment, the real-space patterns of beam 1 and 2 show donut shapes, and the interference pattern exhibits circular dislocation lines. (b) When a polarizer along 30° is placed before the observation, the real-space patterns of beam 1 and 2 show two-lobe patterns. In this case, the dislocation line disappears in the interference pattern, thus making the fork patterns more evident (white arrows).

10 Statistical and robustness analysis of chiral emission

In this section, we discuss the reproducibility of the chiral emission, which is of great significance for practical applications. We fabricated and carried out experiments on 15 samples from the same design to show the statistics. Note that, although the design is identical, the samples would inevitably vary because of fabrication randomness. Namely, the fabrication imperfection would make them randomly and inherently with weak left or right chirality. In the self-interference experiment, we fine-tune the excitation condition to maximize the chiral emission, and the direction of chirality is identified from the orientation of the self-interference fork pattern, while the degree of chirality is solved from the contrast of fork patterns.

The statistical results of the samples are presented in Fig. S13b. Among them, 6 of the 15 samples show considerably high chirality ($|\alpha_{ch}| \geq 0.4$), while 6 samples have relatively weak chirality ($0.4 \geq |\alpha_{ch}| \geq 0$). It is noted that these samples are pumped with the asymmetric spot but at optimized pump positions. Exemplary interference patterns of 3 samples are shown in Fig. S13a, which corresponds to samples with chirality $\alpha_{ch} \leq -0.8$, $\alpha_{ch} = 0$, and $\alpha_{ch} \geq 0.8$, respectively. Among 15 samples, 3 samples did not exhibit chirality after fine-tuning. As discussed above, when the inherent mode splitting is too strong, merely using asymmetric pumping is insufficient to compensate for the inherent couplings for realizing chiral emission.

We also conducted a pump-dependent self-interference measurement to track the evolution of fork-shaped interference patterns. Specifically, we keep the shape and position of the pump beam being fixed and fine-tune the sample position to make the pump beam spot on different positions, as shown in Fig. S14. When the pump beam incidents on the outer area of the sample (left panel), the self-interference pattern shows high fringe contrast, thus indicating high chirality. However, when the spots become closer to the center area of the sample, the fork fringes become vague and even disappear (medium panel and right panel), indicating the reduction and vanishing of the chirality. In this case, the beam profile is distorted and no longer maintains a donut shape. Calibrated from the sample's size, the shifting range of the pump beam is about 10% of the cavity size.

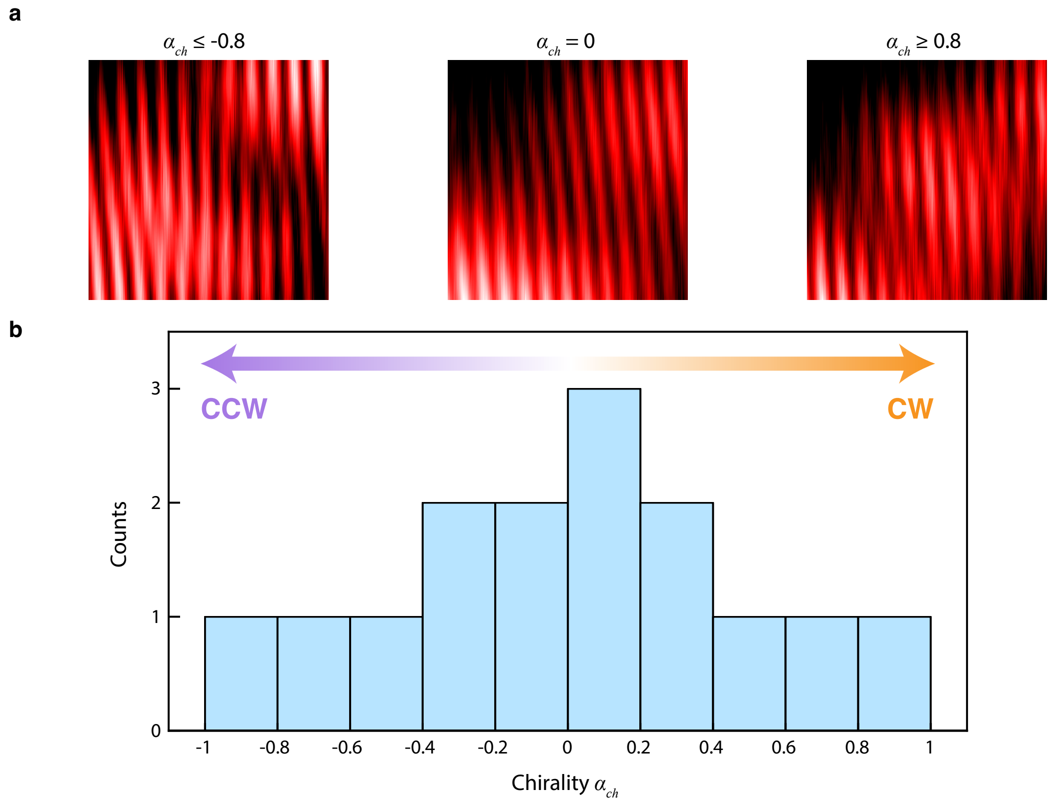

Figure S13: **Statistical analysis of chiral emission for 15 samples with identical design.** (a) The observed exemplary self-interference patterns for different chirality. (b) In the histogram of chiral statistics, 12 of 15 samples exhibit chirality.

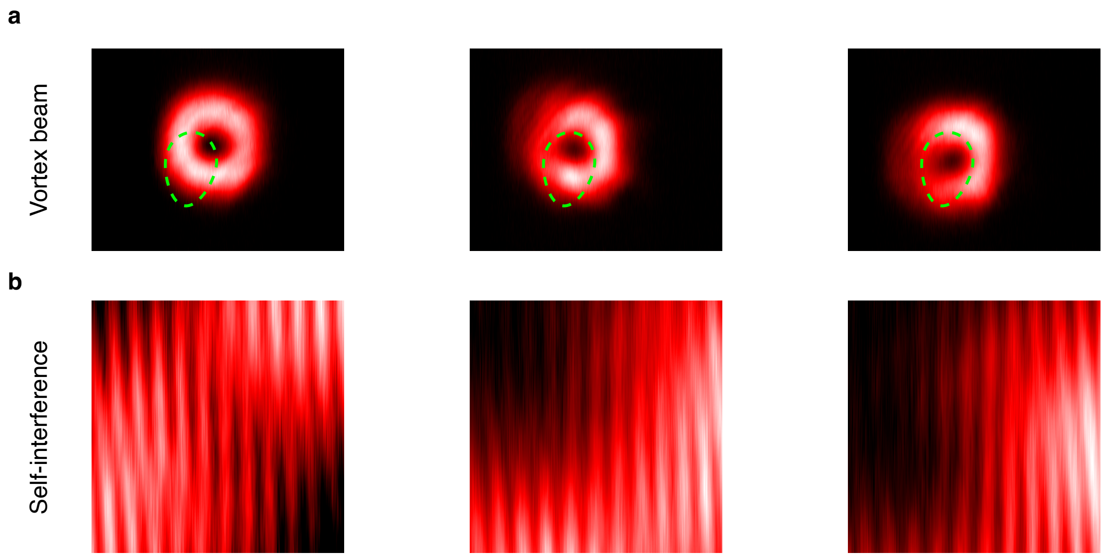

Figure S14: **The evolution of self-interference patterns when the sample is asymmetrically pumped at different positions.** (a) The observed lasing beam patterns in real space. (b) Self-interference patterns in real space, showing the fork pattern (left panel) vanishes (middle panel), and restores (right panel).

References

1. Liang, Y., Peng, C., Sakai, K., Iwahashi, S. & Noda, S. Three-dimensional coupled-wave model for square-lattice photonic crystal lasers with transverse electric polarization: A general approach. *Phys. Rev. B* **84**, 195119 (2011).

2. Peng, C., Liang, Y., Sakai, K., Iwahashi, S. & Noda, S. Coupled-wave analysis for photonic-crystal surface-emitting lasers on air holes with arbitrary sidewalls. *Opt. Express* **19**, 24672–24686 (2011).

3. Liang, Y., Peng, C., Sakai, K., Iwahashi, S. & Noda, S. Three-dimensional coupled-wave analysis for square-lattice photonic crystal surface emitting lasers with transverse-electric polarization: Finite-size effects. *Opt. Express* **20**, 15945–15961 (2012).

4. Peng, C., Liang, Y., Sakai, K., Iwahashi, S. & Noda, S. Three-dimensional coupled-wave theory analysis of a centered-rectangular lattice photonic crystal laser with a transverse-electric-like mode. *Phys. Rev. B* **86**, 035108 (2012).

5. Liang, Y. *et al.* Three-dimensional coupled-wave analysis for triangular-lattice photonic-crystal surface-emitting lasers with transverse-electric polarization. *Opt. Express* **21**, 565–580 (2013).

6. Chen, Z. *et al.* Analytical theory of finite-size photonic crystal slabs near the band edge. *Opt. Express* **30**, 14033–14047 (2022).

7. Chen, Z. *et al.* Observation of miniaturized bound states in the continuum with ultra-high quality factors. *Sci. Bull.* **67**, 359–366 (2022).

8. Ren, Y. *et al.* Low-threshold nanolasers based on miniaturized bound states in the continuum. *Sci. Adv.* **8**, eade8817 (2022).

9. Ni, L., Wang, Z., Peng, C. & Li, Z. Tunable optical bound states in the continuum beyond in-plane symmetry protection. *Phys. Rev. B* **94**, 245148 (2016).

10. Wiersig, J., Kim, S. W. & Hentschel, M. Asymmetric scattering and nonorthogonal mode patterns in optical microspirals. *Phys. Rev. A* **78**, 053809 (2008).

11. Wiersig, J. *et al.* Nonorthogonal pairs of copropagating optical modes in deformed microdisk cavities. *Phys. Rev. A* **84**, 023845 (2011).

12. Wiersig, J. Structure of whispering-gallery modes in optical microdisks perturbed by nanoparticles. *Phys. Rev. A* **84**, 063828 (2011).

13. Kullig, J. & Wiersig, J. Perturbation theory for asymmetric deformed microdisk cavities. *Phys. Rev. A* **94**, 043850 (2016).

14. Peng, B. *et al.* Chiral modes and directional lasing at exceptional points. *Proceedings of the National Academy of Sciences* **113**, 6845–6850 (2016).
