# Supplementary material for: Observation of chiral emission enabled by collective guided resonances
Source: Nat Nanotechnol. 2025 Jul 1;20(9):1205–12. doi: 10.1038/s41565-025-01964-7 (PMC12443609; doi:10.1038/s41565-025-01964-7)
Supplement: Supplementary file 2 — Reporting Summary [file 41565_2025_1964_MOESM2_ESM.pdf]

Corresponding author(s): Wanhua Zheng, Yuri Kivshar and Chao Peng

Lasing Reporting Summary

Nature Research wishes to improve the reproducibility of the work that we publish. This form is intended for publication with all accepted papers reporting claims of lasing and provides structure for consistency and transparency in reporting. Some list items might not apply to an individual manuscript, but all fields must be completed for clarity.

For further information on Nature Research policies, including our data availability policy, see Authors & Referees.

Experimental design

Please check: are the following details reported in the manuscript?

1. Threshold

Plots of device output power versus pump power over a wide range of values indicating a clear threshold

☒ Yes ☐ No — Figure 5 (b) in the main text

2. Linewidth narrowing

Plots of spectral power density for the emission at pump powers below, around, and above the lasing threshold, indicating a clear linewidth narrowing at threshold

☒ Yes ☐ No — Figure 5 (a) in the main text

Resolution of the spectrometer used to make spectral measurements

☒ Yes ☐ No — Section Measurement and data processing of Methods

3. Coherent emission

Measurements of the coherence and/or polarization of the emission

☒ Yes ☐ No — Figure 5 (c) in the main text

4. Beam spatial profile

Image and/or measurement of the spatial shape and profile of the emission, showing a well-defined beam above threshold

☒ Yes ☐ No — The inset of Figure 5 (b) in the main text

5. Operating conditions

Description of the laser and pumping conditions
Continuous-wave, pulsed, temperature of operation

☒ Yes ☐ No — Section Measurement and data processing of Methods

Threshold values provided as density values (e.g. W cm^{-2} or J cm^{-2}) taking into account the area of the device

☒ Yes ☐ No — The caption of Figure 5 (b) in the main text

6. Alternative explanations

Reasoning as to why alternative explanations have been ruled out as responsible for the emission characteristics
e.g. amplified spontaneous, directional scattering; modification of fluorescence spectrum by the cavity

☒ Yes ☐ No — The second paragraph of Section Experimental results in the main text

7. Theoretical analysis

Theoretical analysis that ensures that the experimental values measured are realistic and reasonable
e.g. laser threshold, linewidth, cavity gain-loss, efficiency

☒ Yes ☐ No — Section Principle and design in the main text

8. Statistics

Number of devices fabricated and tested

☒ Yes ☐ No — 15 samples (see Suppl. Section 10 for details)

Statistical analysis of the device performance and lifetime (time to failure)

☒ Yes ☐ No — Suppl. Section 10 (Statistical and robustness analysis of chiral emission)
